# Primary Visual Cortex as a Saliency Map: A Parameter-Free Prediction and Its Test by Behavioral Data

**DOI:** 10.1371/journal.pcbi.1004375

**Published:** 2015-10-06

**Authors:** Li Zhaoping, Li Zhe

**Affiliations:** 1 University College London, London, United Kingdom; 2 Tsinghua University, Beijing, China; Indiana University UNITED STATES

## Abstract

It has been hypothesized that neural activities in the primary visual cortex (V1) represent a saliency map of the visual field to exogenously guide attention. This hypothesis has so far provided only qualitative predictions and their confirmations. We report this hypothesis’ first quantitative prediction, derived without free parameters, and its confirmation by human behavioral data. The hypothesis provides a direct link between V1 neural responses to a visual location and the saliency of that location to guide attention exogenously. In a visual input containing many bars, one of them saliently different from all the other bars which are identical to each other, saliency at the singleton’s location can be measured by the shortness of the reaction time in a visual search for singletons. The hypothesis predicts quantitatively the whole distribution of the reaction times to find a singleton unique in color, orientation, and motion direction from the reaction times to find other types of singletons. The prediction matches human reaction time data. A requirement for this successful prediction is a data-motivated assumption that V1 lacks neurons tuned simultaneously to color, orientation, and motion direction of visual inputs. Since evidence suggests that extrastriate cortices do have such neurons, we discuss the possibility that the extrastriate cortices play no role in guiding exogenous attention so that they can be devoted to other functions like visual decoding and endogenous attention.

## Author Summary

It has been hypothesized that neural activities in the primary visual cortex represent a saliency map of the visual field to exogenously guide attention. This hypothesis has so far provided only qualitative predictions and their confirmations. We report this hypothesis’ first quantitative prediction, derived without free parameters, and its confirmation by human behavioral data. Using the shortness of reaction times in visual search tasks to measure saliency of the search target’s location, the hypothesis predicts the quantitative distribution of the reaction times to find a salient bar unique in color, orientation, and motion direction in a background of bars that are identical to each other. The prediction matches experimental observations in human observers. Since the prediction would be invalid without a particular neural property of the primary visual cortex, the extrastriate cortices may give little contribution to exogenous attentional guidance since they lack this neural property. Implications of this prospect on the framework of attentional network and the computational role of the higher brain areas are also discussed.

## Introduction

### Attentional selection and saliency

Spatial visual selection, often called spatial attentional selection, enables vision to select a visual location for detailed processing using limited cognitive resources [1]. Metaphorically, the selected location is said to be in the attentional spotlight, typically centered on the gaze position. An object outside the spotlight is difficult to recognize. Therefore, the reaction time (RT) to find a particular word on this page depends on how long it takes the spotlight to arrive at the word location. The spotlight is guided by goal-dependent (or top-down, endogenous) mechanisms, such as to direct our gaze to the right words while reading, and/or by goal-independent (or bottom-up, exogenous) mechanisms such as when our reading is distracted by a sudden drastic change in visual periphery.

In this paper, an input is said to be salient when it strongly attracts attention by bottom-up mechanisms, and the degree of this attraction is defined as saliency. For example, an orientation singleton such as a vertical bar in a background of horizontal bars is salient, so is a color singleton such as a red dot among many green ones; and the location of such a singleton has a high saliency value. Therefore, saliency of a visual location can often be measured by the shortness of the reaction time in a visual search to find a target at this location [2], provided that saliency, rather than top-down attention, dictates the variabilities of attentional guidance and reaction time. It can also be measured by attentional (exogenous) cueing effect, the degree in which a salient location speeds up and/or improves visual discrimination of a probe presented at this location immediately after a brief salient cue at the same location [3, 4].

Traditional views presume that higher brain areas, such as those in the parietal and frontal brain areas, are responsible for guiding attention exogenously [2, 5, 6, 1]. This belief was partly inspired by noting that saliency is a general property that could arise from visual inputs with any feature values (e.g., vertical or red) in any feature dimension (e.g., color, orientation, and motion) whereas neurons in lower visual areas like the primary visual cortex are (more likely) tuned to specific feature values (e.g., a vertical orientation) rather than being feature untuned.

### V1 saliency hypothesis: Its feature-blind nature, neural mechanisms, and qualitative experimental support

**Fig 1:**
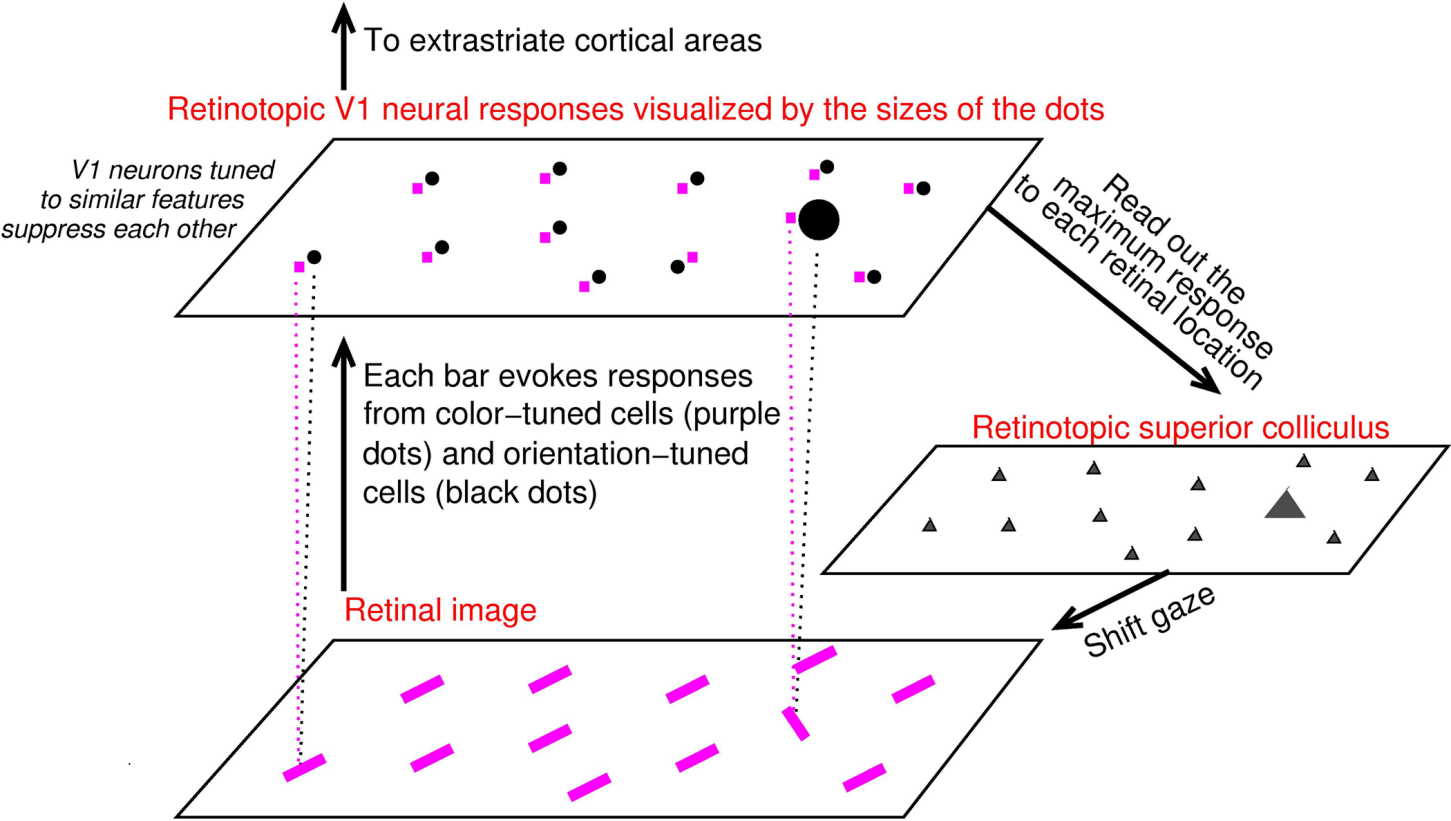
V1 saliency hypothesis states that the bottom-up saliency of a location is represented by the maximum V1 response to this location. In this schematic, V1 is simplified to contain only two kinds of neurons, one tuned to color (their responses are visualized by the purple dots) and the other tuned to orientation (black dots). Each input bar evokes responses in a cell tuned to its color and another cell tuned to its orientation (indicated for two input bars by linking each bar to its two evoked responses by dotted lines), and the receptive fields of these two cells cover the same bar location even though (for better visualization) the dots representing these cells are not overlapping in the cortical map. Iso-feature suppression makes nearby V1 neurons tuned to similar features (e.g., similar color or similar orientation) suppress each other. The orientation singleton in this image evokes the highest V1 response to this image because the orientation-tuned neuron responding to it escapes iso-orientation suppression. The color tuned neuron tuned and responding to the singleton’s color is under iso-color suppression. The saliency map is likely read out by the superior colliculus to execute gaze shifts to salient locations [9].

**Fig 2:**
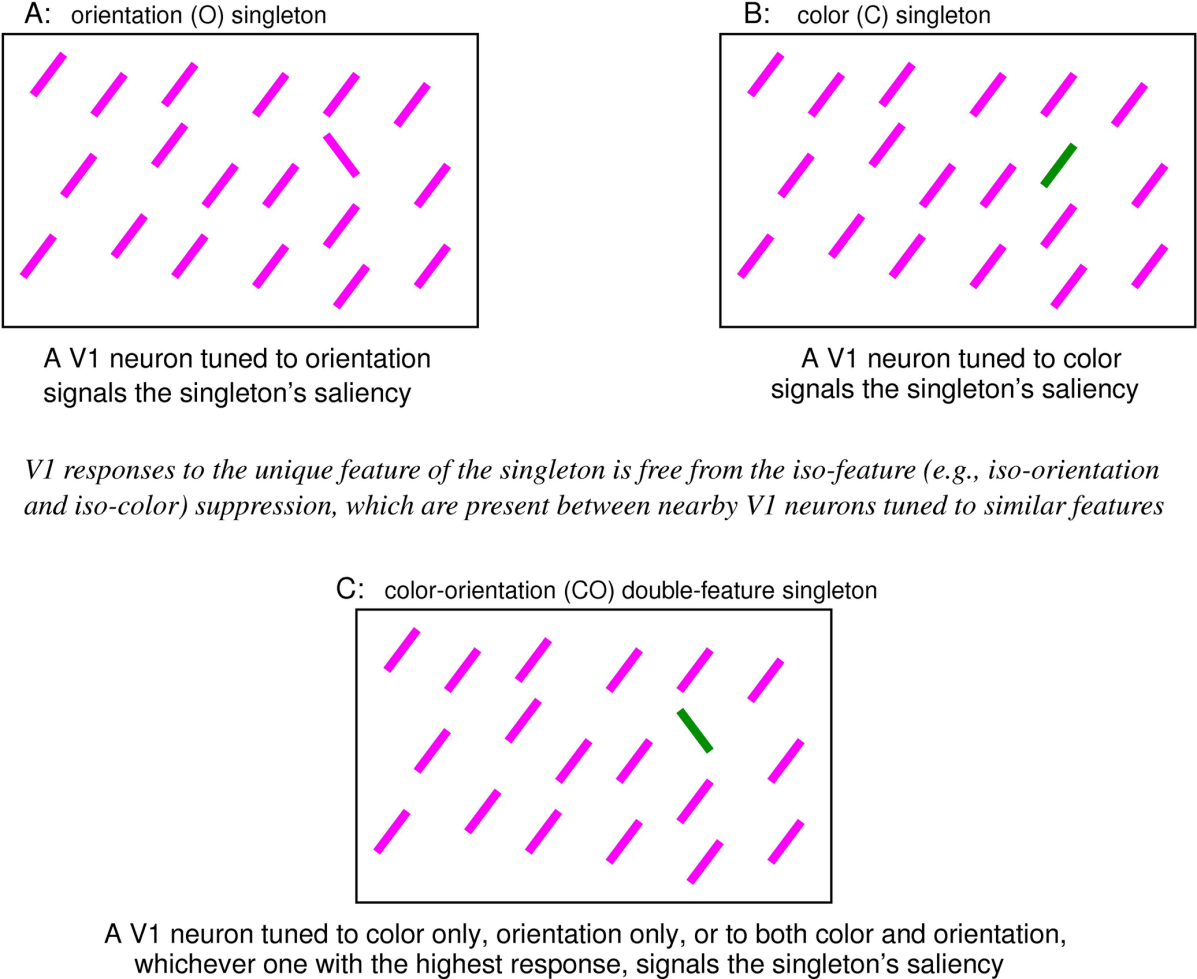
Schematics of visual stimuli for singleton searches. Due to iso-feature suppression, the highest response to each image is from a neuron responding to the singleton bar. This most activated neuron is tuned to orientation for image A, tuned to color for image B, and to color, orientation, or both features of the singleton for image C. The maximum V1 response to the singleton signals the saliency of its location.

It was proposed a decade ago [7, 8] that V1 computes a saliency map, such that the saliency of a location is represented by the maximum response from V1 neurons to this location relative to the maximum responses to the other locations. It is only the V1 response vigor that matters for saliency, and not the preferred features of the responding neurons. For example, the image in Fig 1 contains many colored bars, each activates some V1 neurons tuned to its color and/or orientation. The maximum response to each bar signals the saliency of its location regardless of whether the V1 neuron giving this response is tuned to the color or orientation (or both color and orientation) of the bar. In another example, Fig 2A and 2B contain an orientation and color singleton, respectively, in the same background of uniformly feature bars. If the two images evoke the same background V1 responses to all the background locations, then the two singletons are equally salient if they evoke the same level of maximum response even if the maximum response is evoked in an orientation-tuned cell in one image and a color-tuned cell in the other; conversely, if the two singletons differ by their respective maximally evoked responses, then the singleton evoking the higher response is more salient regardless of the preferred features of the responding neurons.

The feature-blind nature of this saliency representation in V1 enables the brain to have a bottom-up saliency map in V1 in terms of the various maximum V1 responses for various locations, despite the feature tuning of V1 neurons, without resorting to higher cortical areas such as the frontal eye field or lateral-intraparietal cortex [10, 1]. This saliency map may potentially be read out by the superior colliculus, which receives monosynaptic input from V1 and controls eye movement to execute the attentional selection [11]. If an observer searches for a uniquely oriented bar in the retinal image in Fig 1, the reaction time to find this target bar, associated with the saliency of the target location, should thus be associated with the maximum V1 response to this location. In particular, a shorter reaction time should result from a larger value of the maximum response to the target location (when the maximum responses to various non-target locations are fixed).

The neural mechanisms in V1 to compute saliency is intracortical interactions that cause contextual influences, making a neuron’s response to inputs within its receptive field dependent on contextual inputs [12, 13, 14]. One particular form of contextual influences is iso-orientation suppression between nearby neurons tuned to same or similar orientations. It makes orientation-tuned neurons responding to neighboring background bars in Fig 1 suppress each other because they are tuned to the same orientation of these bars, whereas the neuron responding to the orientation singleton escapes such suppression because it is tuned to a very different orientation of the singleton. Hence, the orientation singleton in Fig 1 is the most salient because a V1 neuron, with its receptive field covering the bar, responds more vigorously than any neuron responding to the background bars. Throughout the paper, ‘a neuron responding to a bar’ means the most responsive neuron among a local population of neurons with similar input selectivities responding to this bar regardless of the number of neurons in this local population.

In addition to the orientation feature, V1 neurons are also tuned to other input feature dimensions including color, motion direction, and eye of origin [15, 16]. Hence, each colored bar in the retinal image of Fig 1 evokes not only a response in a cell tuned to its orientation but also another response in another cell tuned to its color (omitting other input features for simplicity), this is indicated by the dotted lines linking the two example input bars and their respective evoked V1 responses. In general, there are many V1 neurons whose receptive fields cover the location of each visual input item (including neurons whose preferred orientations or colors do not match the visual input feature), and only the highest response from these neurons represents the saliency of this location according to the V1 saliency hypothesis (note that this highest response is unlikely to be from a neuron whose preferred feature is not in the input item). In the example of Fig 1, responses from the color-tuned neurons to all bars suffer from iso-color suppression [17], which is analogous to iso-orientation suppression, since all bars have the same color. Focusing on V1 neurons tuned to color only and neurons tuned to orientation only for simplicity, the highest response evoked by the orientation singleton is in the orientation-tuned rather than the color-tuned cell, and this response alone (relative to the maximum responses to the background bars) determines the saliency of the orientation singleton. Later in the paper, the notion that many V1 neurons respond to a single input location or item will be generalized to include neurons tuned to motion direction and neurons jointly tuned to multiple feature dimensions. Determining the highest V1 response to each input location will involve determining which of the many neurons whose receptive fields cover this location has the highest response.

Analogous to iso-orientation suppression and iso-color suppression, iso-motion-direction and iso-ocular-origin suppressions are also present in V1 [12, 13, 14, 18, 19, 20], and we call them iso-feature suppression in general [7]. Accordingly, an input singleton in any of these feature dimensions should be salient (see Fig 2B for a color singleton), since the neuron responding to the unique feature of the singleton escapes the iso-feature suppression from the neurons responding to the uniformly featured background items. This is consistent with known behavioral saliency and has led to the successful prediction of the salient singleton in eye-of-origin [21]. Iso-feature suppression is the dominant form of contextual influences, and it is believed to be mediated by intracortical neural connections [22, 23] linking neurons whose receptive fields are spatially nearby but not necessarily overlapping. A neural circuit model of V1 [24, 25, 7, 26, 27] with its intracortical interactions has successfully explained many prototypical visual search and segmentation examples by using the model responses to predict a saliency map which in turn predicts the relative degrees of ease in the visual behavior associated with the saliencies of the task relevant locations.

Although the V1 saliency hypothesis is a significant departure from traditional psychological theories, it has received substantial experimental support [28, 29, 30, 31, 21, 32, 33], detailed in [9]. In particular, behavioral data confirmed the surprising prediction from this hypothesis that an eye-of-origin singleton (e.g., an item uniquely shown to the left eye among other items shown to the right eye) that is hardly distinctive from other visual inputs can attract attention and gaze qualitatively just like, or quantitatively more strongly than, a salient and highly distinctive orientation singleton does [21, 33]. This finding provides a hallmark of the saliency map in V1 because, cortical neurons, except many in V1, are not tuned to eye-of-origin feature [34, 35], making this feature non-distinctive to perception. Furthermore, behavioral data confirmed that saliency is represented by the maximum rather than the weighted summation or the average of responses to a visual location [30, 29]. Functional magnetic resonance imaging and event related potential measurements also confirmed that, when top-down confounds are avoided or minimized, a salient location evokes brain activations in V1 but not in the parietal and frontal regions [32].

### The current study

So far, predictions and experimental tests of the V1 saliency hypothesis have been *qualitative*. Here, we report its first *quantitative* prediction derived without free parameters. The predicted quantity is the distribution of the reaction times in a visual search for a singleton bar unique simultaneously in color, orientation, and motion direction among uniformly featured background bars. We will derive a precise mathematical relationship between this quantity and the distributions of the reaction times to search for other types of singleton bars, thus enabling us to predict this quantity from the observed reaction times for the other singletons. This mathematical relationship requires, other than the V1 saliency hypothesis, only the following two *qualitative* features in neural physiology: (1) the feature-tuned neural interaction, in particular iso-feature suppression that depends on whether the preferred features of the interacting neurons are similar and causes higher responses to feature singletons, and (2) an assumption motivated by data that V1 does not have neurons tuned simultaneously to color, orientation, and motion direction. It does not depend on other details, e.g., colinear facilitation [36, 37] between V1 neurons and its contrast dependence [38, 39, 20]; otherwise, currently imprecise knowledge of V1 physiology (e.g., its intracortical interactions), which may vary with adaptation state and experience of observers, would have prevented the prediction to be parameter-free.

Furthermore, we show that this prediction quantitatively matches previously collected behavioral data [29]. We develop data analysis methods to obtain the predicted distribution of the reaction times for one type of feature singletons from the observed reaction times for the other types of feature singletons, and compare the predicted quantity with its behavioral counterpart using a custom designed statistical test. We further show that our data have a sufficient statistical power to falsify some spurious predictions that are likely incorrect based on V1 physiology. Since the same set of behavioral data and analysis methods are used to test the spurious predictions and our (non-spurious) prediction, we conclude that our (non-spurious) prediction is confirmed within the resolution provided by the statistical power in our data.

In addition, this paper explores the implications of the experimental confirmation of our quantitative (non-spurious) prediction. We will discuss experimental evidence on whether the extrastriate cortical areas also possess the two required physiological features for the prediction and thus whether they can be excluded from playing a role in saliency. Parts of this work have been presented in abstract form elsewhere [40, 41].

## Results

In this section, we show a direct link between the reaction time to find a visual feature singleton in a homogeneous background (like that in Fig 1) and the highest V1 response to this singleton. From this link, we derive the quantitative prediction and present its experimental test. In this process, we also present some related but spurious predictions that should be violated unless certain conditions on the V1 neural mechanisms hold. These spurious predictions and their tests (falsification) by behavioral data not only provide further insights in the underlying neural mechanisms but also verify that our methods can use our behavioral data to falsify a prediction.

### Linking V1 responses with reaction times

When the effect of top-down attentional guidance is negligible or held constant in a visual search task, a higher saliency at the target location should lead to a shorter reaction time to find the target, by the definition of saliency. In stimuli like those in Fig 2, the feature singletons are assumed as salient enough to dictate immediate attention shifts. The latency of the attentional shift to the singleton is shorter for a more salient singleton. Assuming a fixed additional latency from this attention shift to an observer’s response to report the singleton, then the reaction time for the visual search task, e.g., for the reporting response, is determined by the singleton’s saliency.

Let a visual scene have visual input items at *n* locations *i* = 1, 2, …, *n*, and let *r*_*i*_ be the maximum V1 response evoked by location *i*. Then the saliency of location *i* is determined by *r*_*i*_ relative to the other *r*_*j*_ for *j* ≠ *i*. This is because, according to the V1 saliency hypothesis, saliency read-out process is like an auction for attention, with *r*_*i*_ the bidding price for attention by location *i*, such that the location giving the highest bid is the most likely to win attention [42]. Let us order *i* such that

$$\begin{array}{c} r_{1}\geq r_{2}\geq r_{3}\geq ...\geq r_{n}, \end{array}$$ (1)

then, we can use a function *g*(⋅) to formally describe

$$\begin{array}{c} \text{saliency}\;\text{at}\;\text{the}\;\text{most}\;\text{salient}\;\left(\text{when}\;i=1\right)\;\text{location}=g\left(r_{1}|r_{2},r_{3},...,r_{n}\right). \end{array}$$ (2)

This paper is only concerned with scenes like those in Fig 2, and calls each such scene a feature singleton scene. Such a scene has one feature singleton in a background of many items that are identical to each other, and the singleton is far more salient than any other input item. Then, *r*_1_ is the maximum response to the singleton and is substantially and significantly larger than any *r*_*i*_ for *i* > 1 (e.g., *r*_1_ > 20 spikes/second and *r*_*i*_ < 10 spikes/second for *i* > 1). When *n* is very large (e.g., 660 in the visual stimuli we will use later), we can reasonably expect that *g*(*r*_1_∣*r*_2_, *r*_3_, …) depends on (*r*_2_, *r*_3_, …) mainly through the statistical properties across the *r*_*i*_’s (for *i* > 1) rather than the exact value of each *r*_*i*_. Let the statistical properties be partly characterized by the average $\bar{r}$ and standard deviation *σ* across (*r*_2_, *r*_3_, …, *r*_*n*_); then a singleton with a larger $(r_{1}-\bar{r})/\sigma$, and perhaps also a larger $r_{1}/\bar{r}$, tends to be more salient [7]. More strictly, the function *g*(*r*_1_∣*r*_2_, *r*_3_, …, *r*_*n*_) may also depend on the locations of visual inputs for all *i*. However, we assume that this dependence is negligible in this paper since we are only concerned with singleton scenes satisfying the following condition: (1) the eccentricity of the singleton from the center of the visual field is the same across all singleton scenes, (2) different non-singleton items evoke sufficiently similar maximum responses *r*_*i*_ for *i* > 1, and (3) the distribution of the locations of the non-singleton items is approximately the same across all singleton scenes.

If two scenes are identical to each other in terms of the number *n* of visual input locations and the distribution of the responses *r*_2_, *r*_3_, …, *r*_*n*_, we say that they share an *invariant background response distribution*. The three singleton scenes in Fig 2 are approximately sharing an invariant background response distribution, even though the highest response *r*_1_ to the singleton may be larger in Fig 2C than Fig 2A 2B. This is because the response *r*_*i*_ to each background bar *i* > 1 is determined by the bar itself and by its surrounding neighbors which exert contextual influence (mainly iso-feature suppression) on the response, the singleton can at best be the least influential neighbor since its most activated neuron exerts a limited or negligible iso-feature suppression on neurons most responsive to the background bar and preferring a very different feature. Hence the singleton has a negligible influence on the statistical properties of the background responses, which are determined by such characteristics as the contrast, density, and the degree of regularities in the locations of the background bars.

Assuming an invariant background response distribution shared by a set of feature singleton scenes, we can omit the explicit expression of (*r*_2_, *r*_3_, …) in Eq (2) and write (still using the same notation *g*(⋅) for convenience)

$$\begin{array}{c} \text{the}\;\text{saliency}\;\text{of}\;\text{the}\;\text{singleton}\;\text{location}=g\left(r_{1}\right),r_{1}\;\text{is}\;\text{the}\;\text{highest}\;\text{response}\;\text{to}\;\text{the}\;\text{singleton}. \end{array}$$ (3)

The *g*(*r*) monotonically increases with *r* in a way that is determined by the properties of the invariant background response distribution. Since a larger saliency at the singleton location gives a shorter reaction time to find it (assuming again negligible or constant top-down factors), we can write

$$\begin{array}{c} \text{the}\;\text{reaction}\;\text{time}\;\text{for}\;\text{a}\;\text{feature}\;\text{singleton}=f\left(r_{1}\right),r_{1}\text{is}\;\text{the}\;\text{highest}\;\text{response}\;\text{to}\;\text{the}\;\text{singleton}, \end{array}$$ (4)

and *f*(*r*_1_) is a monotonically decreasing function of *r*_1_. The exact form of *f*(*r*) should depend on the invariant background response distribution, the saliency read-out system, and the observer (e.g., some observers can respond faster than others). We will see that the details of *f*(*r*) do not matter as long as *f*(*r*) monotonically decreases with *r*. With *f*(*r*), the reaction time for a feature singleton is directly linked to its maximum evoked V1 responses.

### A previously known race model in reaction times can be derived from a toy V1

Let us call the singletons in Fig 2A, Fig 2B, and Fig 2C (which share an invariant background response distribution) O, C, and CO singletons, respectively, by the feature dimension(s) in which the singleton has a unique feature. The C and O singletons are single-feature singletons and the CO singleton is a double-feature singleton. Let a toy V1 have only two kinds of neurons, one tuned to color only and one tuned to orientation only, and assume that V1 responses are deterministic rather than stochastic given a visual input. The toy V1 and the deterministic nature of V1 responses are both temporary simplifications to illustrate the method, and these simplifications will be removed later. Let *r* ^*O*^ or *r* ^*C*^, respectively, be the response of the orientation-tuned neuron or the color-tuned neuron to the singleton in Fig 2A or Fig 2B, respectively. They are also the highest responses to the respective singletons due to iso-feature suppression. Then, according to Eq (4), the reaction times *RT*_*O*_ and *RT*_*C*_ to find the O and C singletons, respectively, are

$$\begin{array}{c} {RT}_{O}=f\left(r^{O}\right)\;\text{and}\;{RT}_{C}=f\left(r^{C}\right). \end{array}$$ (5)

The CO singleton in Fig 2C should evoke higher responses in both the neuron tuned to its unique orientation and the neuron tuned to its unique color than the responses to the background bars, again due to iso-feature suppression. Furthermore, we assume that the response property of the orientation-tuned neuron and the contextual influences on it are not affected by the color of the visual input, so that *r* ^*O*^ is the same to the O and CO singletons. Analogously, the response *r* ^*C*^ of the color-tuned neuron is assumed the same to the C and CO singletons. The maximum V1 response to the CO singleton is max(*r* ^*C*^, *r* ^*O*^) (where max(⋅) means the maximum value among the arguments). Hence, the reaction time *RT*_*CO*_ to find the CO singleton is

$$\begin{array}{c} {RT}_{C\;O}=f[\text{max}(r^{C},r^{O})]=\text{min}[f(r^{C}),f(r^{O})]=\text{min}\left({RT}_{C},{RT}_{O}\right), \end{array}$$ (6)

when we combine Eqs (4) and (5) and note that *f*(⋅) is a monotonically decreasing function (min(⋅) means the minimum value of the arguments). The equation

$$\begin{array}{c} {RT}_{C\;O}=\text{min}\left({RT}_{C},{RT}_{O}\right) \end{array}$$ (7)

describes the deterministic version of a race model, often used to model a behavioral reaction time as the shorter reaction time of two or more underlying processes [43], as if (e.g.,) the reaction time for the CO singleton is the winning reaction time in a race between two racers, C and O singletons, with their respective reaction times. Here we see (see also [29]) that this model can arise from the neural substrates, given the V1 saliency hypothesis, if V1 has only neurons tuned to orientation only and neurons tuned to color only but no neurons tuned to both. This is because by such a V1 the double-feature singleton is as salient as the more salient of the two single-feature singletons. We note that this race model arises regardless of the details of *f*(*r*) as long as it is a monotonically decreasing function.

V1 responses are actually stochastic, each a random sample from a specific distribution. To proceed, we assume the following two conditions. First, there are sufficiently many background items that the statistical properties of the invariant background response distribution (e.g., the mean and standard deviation across the responses to the background items) are not stochastic despite the stochasticity of the individual responses. Second, the singletons are salient enough that their evoked responses *r* ^*C*^ and *r* ^*O*^ are always larger than any responses to the background. By Eq (5), the stochastic *r* ^*C*^ and *r* ^*O*^ make *RT*_*C*_ and *RT*_*O*_ also stochastic. For example, if *P*_*r*^*O*^_(*r* ^*O*^) is the probability density of *r* ^*O*^, then the probability density of *RT*_*O*_ is

$$\begin{array}{c} P_{{RT}_{O}}\left({RT}_{O}\right)=[P_{r^{O}}\left(r^{O}\right){\left(\frac{df(r^{O})}{dr^{O}}\right)}^{-1}],\;\text{at}\;r^{O}=f^{-1}\left({RT}_{O}\right). \end{array}$$ (8)

In any case, *RT*_*CO*_ = *f*[max(*r* ^*C*^, *r* ^*O*^)] = min[*f*(*r* ^*C*^), *f*(*r* ^*O*^)] still holds. If the trial to trial fluctuations of *r* ^*C*^ and *r* ^*O*^ are regardless of the visual input in the feature dimension in which the neuron is untuned, and if they fluctuate independently of each other in the responses to the CO singleton, then the deterministic equation *RT*_*CO*_ = min(*RT*_*C*_, *RT*_*O*_) becomes

$$\begin{array}{c} \text{Distribution}\;\text{of}\;{RT}_{C\;O}=\text{Distribution}\;\text{of}\;\;\text{min}\left({RT}_{C},{RT}_{O}\right), \end{array}$$ (9)

in which *RT*_*C*_ and *RT*_*O*_ are independent random samples from their respective distributions. The average of *RT*_*CO*_ will be shorter than *both* the average of *RT*_*C*_ *and* the average of *RT*_*O*_, due to statistical facilitation, since each sample of *RT*_*CO*_ is the race winner of the two samples *RT*_*C*_ and *RT*_*O*_. For simplicity, Eq (9) is written by this shorthand

$$\begin{array}{c} {RT}_{C\;O}\;\overset{P}{=}\;\text{min}\left({RT}_{C},{RT}_{O}\right), \end{array}$$ (10)

with $x\;\overset{P}{=}\;y$ to mean that *x* and *y* have the same probability distribution.

The race model, or race equality, ${RT}_{C\;O}\overset{P}{=}\text{min}({RT}_{C},{RT}_{O})$ is a prediction of the V1 saliency hypothesis if one were hypothetically to assume a toy V1 that has no V1 neuron which can respond more vigorously to the CO singleton than the orientation-only-tuned neuron and the color-only-tuned neuron. This assumption is wrong. Hence ${RT}_{C\;O}\overset{P}{=}\text{min}({RT}_{C},{RT}_{O})$ is called a spurious race equality and its predicted distribution of *RT*_*CO*_ from experimentally observed distribution of min(*RT*_*C*_, *RT*_*O*_) is called a spurious prediction.

### The spurious race equality ${RT}_{C\;O}\overset{P}{=}\text{min}({RT}_{C},{RT}_{O})$ is violated

**Fig 3:**
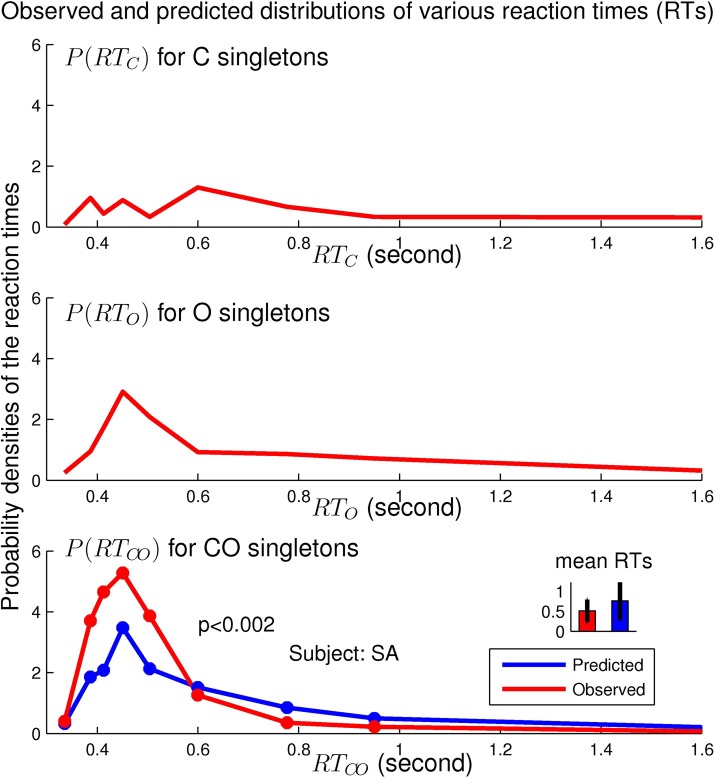
Behavioral refutation of a spurious prediction ${RT}_{C\;O}\overset{P}{=}\text{min}({RT}_{C},{RT}_{O})$ based on the incorrect assumption that V1 lacks neurons tuned simultaneously to both orientation and color. The graphs show distributions (in discrete time bins) of *RT*_*O*_, *RT*_*C*_, and *RT*_*CO*_ (and the average and the standard deviation of *RT*_*CO*_) of a particular observer SA in searches of the singletons. Experimental data are shown in red, the prediction is in blue. The predicted and actual distributions of *RT*_*CO*_ are significantly different from each other, indicated by a *p* < 0.002 in the bottom plot.

Fig 3 shows that the spurious prediction of the distribution of *RT*_*CO*_ is significantly different from the distribution of the behaviorally observed *RT*_*CO*_, with a *p* value *p* < 0.002 in the statistical test of the null hypothesis that the predicted and the observed distributions of *RT*_*CO*_ are the same. (See the Methods section for how to obtain the prediction and the *p* value). The behavioral *RT*_*CO*_ values are significantly shorter than the predicted ones.

**Fig 4:**
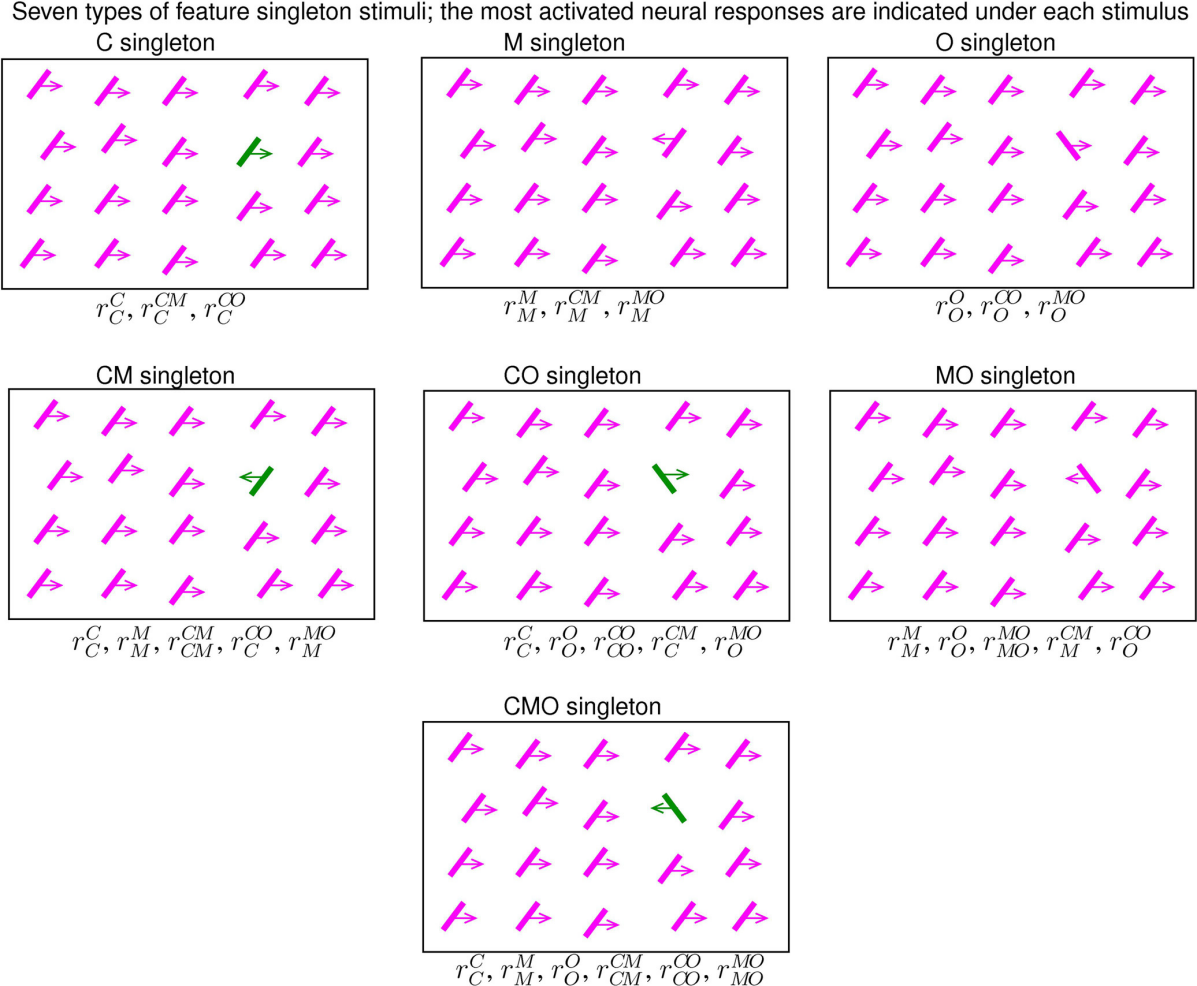
Schematics of the seven kinds of feature singleton scenes. Each bar is colored green or purple (of the same luminance in the behavioral experiment), tilted to the left or right from vertical by the same absolute tilt angle, moving to the left or right (indicated by an arrow pointing to left or right) by the same motion speed. Under each schematic, the non-trivial neural responses (e.g., these responses are expected to be substantially higher than the responses to the background bars) evoked by the singleton are listed. Each singleton scene here is called a purple scene (in this paper) to denote that the color of the background bars are purple. Swapping between the green and purple colors changes a purple scene into a green scene. All purple scenes are assumed to share an invariant background response distribution, so are all the green scenes. The behavioral experiment [29] randomly interleaved purple and green scenes between trials.

With motion direction as another feature dimension, a feature singleton in motion direction, an M singleton, is the analogy of a C or O singleton. Analogous to a CO singleton, a double-feature singleton CM or MO is unique in both color and motion direction, or in both motion direction and orientation, respectively. A triple-feature CMO singleton is unique in all the three feature dimensions. Fig 4 shows the schematics of all the seven types of singletons. Let the reaction times to find singletons M, CM, MO, and CMO be *RT*_*M*_, *RT*_*CM*_, *RT*_*MO*_, and *RT*_*CMO*_, respectively. Then the spurious equality ${RT}_{C\;O}\;\overset{P}{=}\;\text{min}({RT}_{C},{RT}_{O})$ has the following generalizations:

$$\begin{array}{c} {RT}_{C\;M}\overset{P}{=}\text{min}\left({RT}_{C},{RT}_{M}\right), \end{array}$$ (11)

$$\begin{array}{ccc} {RT}_{M\;O} & \overset{P}{=} & \text{min}({RT}_{M},{RT}_{O}),\;\text{and} \end{array}$$ (12)

$$\begin{array}{ccc} {RT}_{C\;M\;O} & \overset{P}{=} & \text{min}({RT}_{C},{RT}_{M},{RT}_{O}). \end{array}$$ (13)

**Fig 5:**
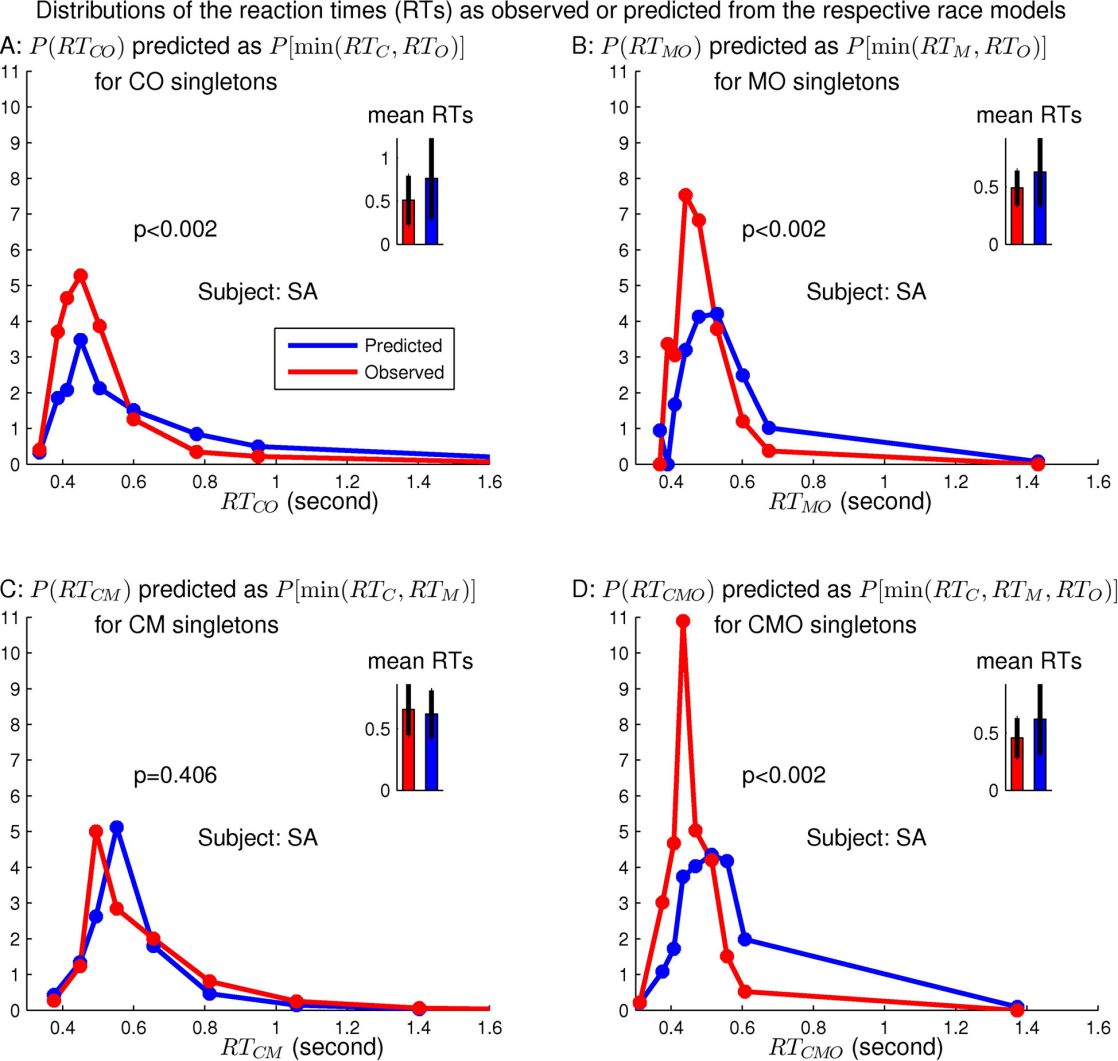
The observed and predicted distributions of reaction times for a double- or triple-feature singleton, using four different race models (race equalities), ${RT}_{C\;O}\overset{P}{=}\text{min}({RT}_{C},{RT}_{O})$ (in panel A), ${RT}_{M\;O}\overset{P}{=}\text{min}({RT}_{M},{RT}_{O})$ (in panel B), ${RT}_{C\;M}\overset{P}{=}\text{min}({RT}_{C},{RT}_{M})$ (in panel C), or ${RT}_{C\;M\;O}\overset{P}{=}\text{min}({RT}_{C},{RT}_{M},{RT}_{O})$ (in panel D), in a race between the corresponding racers whose reaction times are those of the corresponding single-feature singletons. The data are from the same subject SA already shown in Fig 3, panel A shows the same information as that in the bottom panel of Fig 3. The predicted and observed distributions are significantly different from each other except in panel C.

Each equality above holds when V1 is assumed to have no neurons, i.e., the CM, MO, CO, or CMO neurons, which are tuned to more than one feature dimension and can respond more vigorously to the corresponding double-feature (or triple-feature) singleton than it does to the corresponding singleton-feature singletons. Each equality predicts the distribution of the reaction times for a double- or triple-feature singleton from the observed reaction times for the corresponding single-feature singletons. Using data from the same observer as that in Fig 3, Fig 5 shows that other than *RT*_*CM*_, the predictions disagree with the behavioral observations.

### V1 neurons tuned conjunctively to color and orientation predict that *RT*_*CO*_ is likely shorter than predicted by the race model

Here we show that, because real V1 contains neurons (we call CO neurons) that are tuned simultaneously to color and orientation [16], the predicted *RT*_*CO*_ using $RT_{C\;O}\overset{P}{=}\text{min}(RT_{C},RT_{O})$ can be longer than the observed *RT*_*CO*_. Neurons tuned to color or orientation only are referred to as C or O neurons. Let *r* ^*CO*^ denote the response of the CO neuron to the CO singleton, which thus evokes a maximum response max(*r* ^*C*^, *r* ^*O*^, *r* ^*CO*^). According to Eq (4),

$$\begin{array}{c} {RT}_{C\;O}=f[\text{max}(r^{C},r^{O},r^{C\;O})]. \end{array}$$ (14)

A CO neuron also responds to a C or O singleton matching its preferred color and orientation. For example, each of the C, O, and CO singletons in Fig 4 evokes a vigorous response in a CO neuron preferring its color and orientation. We use $r_{\alpha }^{C\;O}$ to denote such a response of a CO neuron to a singleton *α* = *C*, *O*, or *C* *O*. Then $r_{C}^{C\;O}$ suffers from iso-orientation suppression (since the C singleton has the same orientation as the background bars), $r_{O}^{C\;O}$ suffers from iso-color suppression, and $r_{C\;O}^{C\;O}$ is free from iso-feature suppressions. For completeness, $r_{B}^{C\;O}$ denotes a CO neuron’s response to a background bar matching its preferred features. Since $r_{B}^{C\;O}$ suffers from both iso-color and iso-orientation suppressions it is likely that $r_{B}^{C\;O}<r_{\alpha }^{C\;O}$ for *α* = *C*, *O*, and *C* *O*.

Our notations for the responses ignore the binary tilt direction (clockwise or anticlockwise from vertical), color (isoluminant purple or green), or motion direction (leftward or rightward) of our singletons. This is because, in terms of evoked V1 response levels under contextual influences, reflection symmetry is assumed between the two tilt directions and between the two motion directions in our singleton scenes (all bars in all scenes have the same absolute angle from vertical and the same absolute motion speed). If a symmetry for V1 responses is not assumed between the two isoluminant colors with associated contextual influences, then our notations and derivations are only applicable when all singleton scenes are restricted to those with a given (e.g., purple) color of the background bars. For convenience, we call our singleton scenes with purple or green background bars purple or green scenes, respectively. For example, all the scenes in Fig 4 are purple. Without the color symmetry, the behavioral data from the purple scenes should be analyzed separately from those from the green scenes.

For consistency, we similarly use $r_{\alpha }^{C}$ and $r_{\alpha }^{O}$ to denote C and O neural responses to a singleton bar *α* = *C*, *O*, and *C* *O* or a background bar *α* = *B*. For example, the responses of the C neuron to the four kinds of bars are $r_{C}^{C}$, $r_{O}^{C}$, $r_{C\;O}^{C}$, and $r_{B}^{C}$. We have previously ignored $r_{O}^{C}$ and identified $r_{C\;O}^{C}$ with $r_{C}^{C}$ since we argued that

$$\begin{array}{c} r_{B}^{C}\;\overset{P}{=}\;r_{O}^{C}\;\text{with}\;\text{iso-color}\;\text{suppression,}\;\text{and}\;r_{C}^{C}\;\overset{P}{=}\;r_{C\;O}^{C}\;\text{without}\;\text{iso-color}\;\text{suppression}, \end{array}$$ (15)

because a C neuron’s response should be regardless of the orientation feature. Similarly, the O neuron’s response should be regardless of the input color (since the green and purple bars have the same high luminance contrast against a dark background) and have the following two types of responses,

$$\begin{array}{c} r_{B}^{O}\;\overset{P}{=}\;r_{C}^{O}\;\text{and}\;r_{O}^{O}\;\overset{P}{=}\;r_{C\;O}^{O}. \end{array}$$ (16)

Neural responses such as $r_{O}^{C}\;(\overset{P}{=}\;r_{B}^{C})$ and $r_{C}^{O}\;(\overset{P}{=}\;r_{B}^{O})$ that can be statistically equated with the same neurons’ responses to a background bar will be called trivial responses.

Note that the meaning of, e.g., *C*, in our mathematical expression depends on whether it is a superscript or a subscript. As a superscript in, e.g., *r* ^*C*^ it means that the neuron giving the response is tuned to the color (C) feature; as a subscript in, e.g., $r_{C}^{O}$ or *RT*_*C*_ it means the input bar evoking the response or reaction time is a color (C) singleton. Without loss of validity, responses from neurons preferring feature(s) different from the feature(s) of the bars are ignored, since they are always smaller and do not affect saliency dictated by the maximum response to each location.

Combining Eq (4) with the equations above, we have

$$\begin{array}{ccc} {RT}_{C} & = & f[\text{max}(r_{C}^{C},r_{C}^{O},r_{C}^{C\;O})]\;\overset{P}{=}\;f[\text{max}(r_{C}^{C},r_{B}^{O},r_{C}^{C\;O})], \end{array}$$ (17)

$$\begin{array}{ccc} {RT}_{O} & = & f[\text{max}(r_{O}^{C},r_{O}^{O},r_{O}^{C\;O})]\;\overset{P}{=}\;f[\text{max}(r_{B}^{C},r_{O}^{O},r_{O}^{C\;O})], \end{array}$$ (18)

$$\begin{array}{ccc} {RT}_{C\;O} & = & f[\text{max}(r_{C\;O}^{C},r_{C\;O}^{O},r_{C\;O}^{C\;O})]\;\overset{P}{=}\;f[\text{max}(r_{C}^{C},r_{O}^{O},r_{C\;O}^{C\;O})]. \end{array}$$ (19)

Since a C singleton is more salient than a background bar, by V1 saliency hypothesis, its maximum evoked response $\text{max}\left(r_{C}^{C},r_{B}^{O},r_{C}^{C\;O}\right)$ must be larger than the maximum response $\text{max}\left(r_{B}^{C},r_{B}^{O},r_{B}^{C\;O}\right)$ to a background bar, i.e., $\text{max}\left(r_{C}^{C},r_{B}^{O},r_{C}^{C\;O}\right)>\text{max}\left(r_{B}^{C},r_{B}^{O},r_{B}^{C\;O}\right)$. Combining this with $\text{max}\left(r_{B}^{C},r_{B}^{O},r_{B}^{C\;O}\right)\geq r_{B}^{O}$ gives $\text{max}\left(r_{C}^{C},r_{B}^{O},r_{C}^{C\;O}\right)>r_{B}^{O}$, consequently $\text{max}\left(r_{C}^{C},r_{B}^{O},r_{C}^{C\;O}\right)=\text{max}\left(r_{C}^{C},r_{C}^{C\;O}\right)$. Similarly $\text{max}\left(r_{B}^{C},r_{O}^{O},r_{O}^{C\;O}\right)=\text{max}\left(r_{O}^{O},r_{O}^{C\;O}\right)$. Hence, we can ignore $r_{B}^{C}$ and $r_{B}^{O}$ in Eqs (17)–(18) to have

$$\begin{array}{c} {RT}_{C}=f[\text{max}(r_{C}^{C},r_{C}^{C\;O})]\;\text{and}\;{RT}_{O}=f[\text{max}(r_{O}^{O},r_{O}^{C\;O})]. \end{array}$$ (20)

The above two equations are just examples of the following equation for our singleton scenes:

$$\begin{array}{c} \text{reaction}\;\text{time}\;\text{to}\;\text{a}\;\text{singleton}=f[\text{max}(\text{list}\;\text{of}\;\text{all}\;\text{the}\;\text{non-trivial}\;\text{responses}\;\text{to}\;\text{this}\;\text{singleton})]. \end{array}$$ (21)

This can be seen by noting that a $r_{C}^{O}\;(\overset{P}{=}\;r_{B}^{O})$ is a trivial response (i.e., statistically the same as the neuron’s response to a background bar) to a C singleton whereas $r_{O}^{C}\;(\overset{P}{=}\;r_{B}^{C})$ is a trivial response to an O singleton. From Eq (20),

$$\begin{array}{ccc} \text{min}({RT}_{C},{RT}_{O}) & = & \text{min}\{f[\text{max}(r_{C}^{C},r_{C}^{C\;O})],f[\text{max}(r_{O}^{O},r_{O}^{C\;O})]\}\\ & = & f\{\text{max}[\text{max}(r_{C}^{C},r_{C}^{C\;O}),\text{max}(r_{O}^{O},r_{O}^{C\;O})]\}\\ & = & f[\text{max}(r_{C}^{C},r_{O}^{O},r_{C}^{C\;O},r_{O}^{C\;O})], \end{array}$$ (22)

in which the second line follows from that *f*(⋅) is a monotonically decreasing function, the third line arises from the equality max(max(*a*, *b*), max(*c*, *d*), …) = max(*a*, *b*, *c*, *d*, …). Eq (22) is a special case of

$$\begin{array}{ccc} & & \text{min}(\text{list}\;\text{of}\;\text{reaction}\;\text{times}\;\text{for}\;\text{various}\;\text{singletons})\\ & & \;=f[\text{max}(\text{list}\;\text{of}\;\text{all}\;\text{the}\;\text{non-trivial}\;\text{responses}\;\text{to}\;\text{these}\;\text{singletons})]. \end{array}$$ (23)

This equation is the extension of Eq (21) to multiple reaction times for multiple singletons, each alone in a singleton scene. It will be used to derive other race equalities.

Since $\text{min}({RT}_{C},{RT}_{O})=f\left[\text{max}\left(r_{C}^{C},r_{O}^{O},r_{C}^{C\;O},r_{O}^{C\;O}\right)\right]$ and $RT_{C\;O}=f\left[\text{max}\left(r_{C}^{C},r_{O}^{O},r_{C\;O}^{C\;O}\right)\right]$, equality ${RT}_{C\;O}\overset{P}{=}\text{min}({RT}_{C},{RT}_{O})$ requires $\text{max}\left(r_{C}^{C},r_{O}^{O},r_{C\;O}^{C\;O}\right)\overset{P}{=}\text{max}\left(r_{C}^{C},r_{O}^{O},r_{C}^{C\;O},r_{O}^{C\;O}\right)$. This requirement can be met either when the CO neural responses are relatively negligible such that

$$\begin{array}{c} \text{max}(r_{C}^{C\;O},r_{O}^{C\;O},r_{C\;O}^{C\;O})<\text{max}(r_{C}^{C},r_{O}^{O}), \end{array}$$ (24)

so as to reduce both $\text{max}\left(r_{C}^{C},r_{O}^{O},r_{C\;O}^{C\;O}\right)$ and $\text{max}\left(r_{C}^{C},r_{O}^{O},r_{C}^{C\;O},r_{O}^{C\;O}\right)$ to $\text{max}\left(r_{C}^{C},r_{O}^{O}\right)$, or

$$\begin{array}{c} r_{C\;O}^{C\;O}\;\overset{P}{=}\;\text{max}(r_{C}^{C\;O},r_{O}^{C\;O}). \end{array}$$ (25)

The two conditions, Eqs (24) and (25), can both be satisfied when CO neurons are absent so that $r_{C}^{C\;O}=r_{O}^{C\;O}=r_{C\;O}^{C\;O}=0$. In this paper, a prediction (such as ${RT}_{C\;O}\overset{P}{=}\text{min}({RT}_{C},{RT}_{O})$) is called spurious if the neural properties (such as the two conditions above) upon which it relies are either known to be violated in V1 or whose presence in V1 is uncertain. Whether the neural properties required for a spurious prediction can be satisfied may depend on individual observers, for example, sensitivities to different colors vary by a few fold between different observers with normal color vision [44] and V1 properties may vary accordingly [45].

Meanwhile, the equality ${RT}_{C\;O}\overset{P}{=}\text{min}({RT}_{C},{RT}_{O})$ is likely broken when the CO neurons are present [16]. Iso-feature suppression makes it likely that

$$\begin{array}{c} \langle r_{C\;O}^{C\;O}\rangle >\langle \text{max}(r_{C}^{C\;O},r_{O}^{C\;O})\rangle , \end{array}$$ (26)

where ⟨*x*⟩ means the ensemble average of *x*. If so, ${RT}_{C\;O}\;\overset{P}{=}\;\text{min}({RT}_{C},{RT}_{O})$ is likely replaced by a race inequality

$$\begin{array}{c} \left\langle {RT}_{C\;O}\right\rangle <\langle \text{min}({RT}_{C},{RT}_{O})\rangle . \end{array}$$ (27)

Hence, the V1 saliency hypothesis predicts *qualitatively* that *RT*_*CO*_ and min(*RT*_*C*_, *RT*_*O*_) are likely to be statistically different, in particular it predicts that *RT*_*CO*_ is likely shorter, without predicting the *quantitative* difference between *RT*_*CO*_ and min(*RT*_*C*_, *RT*_*O*_).

Similarly, V1 also contains MO neurons that are tuned simultaneously to orientation and motion direction [34]. Hence, ${RT}_{M\;O}\;\overset{P}{=}\;\text{min}({RT}_{M},{RT}_{O})$ is likely broken and the following inequality

$$\begin{array}{c} \left\langle {RT}_{M\;O}\right\rangle <\left\langle \text{min}\left({RT}_{M},{RT}_{O}\right)\right\rangle , \end{array}$$ (28)

analogous to ⟨*RT*_*CO*_⟩ < ⟨min(*RT*_*C*_, *RT*_*O*_)⟩, is likely. However, V1 is reported to contain few CM neurons that are tuned simultaneously to color and motion direction [46], although conflicting reports [46, 47, 48] make it unclear whether CM neurons are indeed absent or just fewer. Hence, it is unclear whether ${RT}_{C\;M}\;\overset{P}{=}\;\text{min}({RT}_{C},{RT}_{M})$ holds or whether the inequality ⟨*RT*_*CM*_⟩ < ⟨min(*RT*_*C*_, *RT*_*M*_)⟩ may occur.

Although V1 has CO and MO cells, we do not know enough about their properties. Hence, our educated guesses such as $\left\langle r_{C\;O}^{C\;O}\right\rangle >\left\langle \text{max}\left(r_{C}^{C\;O},r_{O}^{C\;O}\right)\right\rangle$ and the breaking of ${RT}_{C\;O}\;\overset{P}{=}\;\text{min}({RT}_{C},{RT}_{O})$ are merely predicted as likely rather than certain. For observer SA in Fig 5, the behaviorally observed ⟨*RT*_*CO*_⟩ and ⟨*RT*_*MO*_⟩ are indeed shorter than their respective race model predicted values ⟨min(*RT*_*C*_, *RT*_*O*_)⟩ and ⟨min(*RT*_*M*_, *RT*_*O*_)⟩, respectively. Meanwhile, ${RT}_{C\;M}\overset{P}{=}\text{min}({RT}_{C},{RT}_{M})$ holds for this observer within the resolution provided by our data.

The inequality ⟨*RT*_*αα*′_⟩ < ⟨min(*RT*_*α*_, *RT*_*α*′_)⟩ for *α* or *α*′ = *C*, *M*, or *O* and *α* ≠ *α*′ is called a double-feature advantage or redundancy gain, and has been observed previously. Focusing on the time bins for the shortest reaction times, Krummenacher et al [49] showed that the densities of *RT*_*CO*_ in these bins were more than the summations of the densities of the racers *RT*_*C*_ and *RT*_*O*_. Koene and Zhaoping [29] showed that ⟨*RT*_*CO*_⟩ < ⟨min(*RT*_*C*_, *RT*_*O*_)⟩ and ⟨*RT*_*MO*_⟩ < ⟨min(*RT*_*M*_, *RT*_*O*_)⟩ hold statistically across eight observers, whereas the average ⟨*RT*_*CM*_⟩ is not significant different from ⟨min(*RT*_*C*_, *RT*_*M*_)⟩. The current work extends the previous findings by comparing the whole distribution of the observed *RT*_*αα*′_ with that of min(*RT*_*α*_, *RT*_*α*′_). The difference between *RT*_*αα*′_ and min(*RT*_*α*_, *RT*_*α*′_) should reflect the contribution of the double-feature tuned neurons, CO, MO, or CM, to the saliency of the double-feature singleton (via its response $r_{C\;O}^{C\;O}$, $r_{M\;O}^{M\;O}$, or $r_{C\;M}^{C\;M}$, respectively, beyond the contribution of these neurons to the saliency of the single-feature singletons), as evaluated by Zhaoping and Zhe [50].

Generalizing our derivations (in Eqs (14)–(27)), the triple-feature race model $RT_{C\;M\;O}\overset{P}{=}\text{min}(RT_{C},RT_{M},RT_{O})$ is likely broken when the responses from the CM, CO, and MO neurons are not negligible unless, analogous to Eq (25), the response equality $\text{max}\left(r_{C\;M}^{C\;M},r_{C\;O}^{C\;O},r_{M\;O}^{M\;O}\right)\overset{P}{=}\text{max}\left(r_{C}^{C\;M},r_{M}^{C\;M},r_{C}^{C\;O},r_{O}^{C\;O},r_{M}^{M\;O},r_{O}^{M\;O}\right)$ holds. Here, $r_{\alpha }^{C\;M}$ and $r_{\alpha }^{M\;O}$ are responses of the CM and MO neurons, respectively to single- or double-feature singleton *α*, and V1 is assumed to have no CMO cells tuned simultaneously in all the three feature dimensions. Additionally, just as ⟨*RT*_*CO*_⟩ < ⟨min(*RT*_*C*_, *RT*_*O*_)⟩ can arise from $\langle r_{C\;O}^{C\;O}\rangle >\left\langle \text{max}\left(r_{C}^{C\;O},r_{O}^{C\;O}\right)\right\rangle$, the inequality ⟨*RT*_*CMO*_⟩ < ⟨min(*RT*_*C*_, *RT*_*M*_, *RT*_*O*_)⟩ can arise from

$$\begin{array}{c} \langle \text{max}(r_{C\;M}^{C\;M},r_{C\;O}^{C\;O},r_{M\;O}^{M\;O})\rangle >\langle \text{max}(r_{C}^{C\;M},r_{M}^{C\;M},r_{C}^{C\;O},r_{O}^{C\;O},r_{M}^{M\;O},r_{O}^{M\;O})\rangle , \end{array}$$ (29)

which can occur when the double-feature tuned neurons respond more vigorously to the double- or triple-feature singletons than to the single-feature singletons due to iso-feature suppression.

The above inequality is a composite of the three component inequalities $\left\langle r_{C\;O}^{C\;O}\rangle >\langle \text{max}(r_{C}^{C\;O},r_{O}^{C\;O})\right\rangle$, $\left\langle r_{M\;O}^{M\;O}\rangle >\langle \text{max}(r_{M}^{M\;O},r_{O}^{M\;O})\right\rangle$, and $\left\langle r_{C\;M}^{C\;M}\rangle >\langle \text{max}(r_{C}^{C\;M},r_{M}^{C\;M})\right\rangle$. Hence, it is likely to hold when two out of the three component inequalities hold. According to analysis around Eqs (25)–(27), $\left\langle r_{\alpha \;\alpha^{\prime }}^{\alpha \;\alpha^{\prime }}\rangle >\langle \text{max}(r_{\alpha }^{\alpha \;\alpha^{\prime }},r_{\alpha^{\prime }}^{\alpha \;\alpha^{\prime }})\right\rangle$ is implied by race inequality ⟨*RT*_*αα*′_⟩ < ⟨min(*RT*_*α*_, *RT*_*α*′_)⟩ for *α* *α*′ = *C* *O*, *M* *O*, or *C* *M*. Therefore, the triple-racer inequality ⟨*RT*_*CMO*_⟩ < ⟨min(*RT*_*C*_, *RT*_*M*_, *RT*_*O*_)⟩ is quite likely when two out of the three double-racer inequalities ⟨*RT*_*αα*′_⟩ < ⟨min(*RT*_*α*_, *RT*_*α*′_)⟩ hold. This is the case in Fig 5. Meanwhile, the composite equality $\text{max}\left(r_{C\;M}^{C\;M},r_{C\;O}^{C\;O},r_{M\;O}^{M\;O}\right)\overset{P}{=}\text{max}\left(r_{C}^{C\;M},r_{M}^{C\;M},r_{C}^{C\;O},r_{O}^{C\;O},r_{M}^{M\;O},r_{O}^{M\;O}\right)$ may still hold when $r_{\alpha \;\alpha^{\prime }}^{\alpha \;\alpha^{\prime }}\overset{P}{=}\text{max}\left(r_{\alpha }^{\alpha \;\alpha^{\prime }},r_{\alpha^{\prime }}^{\alpha \;\alpha^{\prime }}\right)$ is broken for each component *α* *α*′ = *C* *O*, *M* *O*, and *C* *M*.

### A quantitative prediction of the reaction time for a triple-feature singleton from another race equality

To make a quantitative prediction, we can confidently assume that V1 has no CMO neurons tuned simultaneously to all the three features, C, M, and O, given the existing paucity of neurons tuned simultaneously to C and M [46] (since a CMO neuron should at least be tuned to C and M simultaneously). Just as the absence of CO neurons gives ${RT}_{C\;O}\;\overset{P}{=}\;\text{min}({RT}_{C},{RT}_{O})$, the absence of the CMO neurons gives (see proof in the Method section)

$$\begin{array}{c} \text{min}\left({RT}_{C\;M\;O},{RT}_{C},{RT}_{M},{RT}_{O}\right)\overset{P}{=}\text{min}\left({RT}_{C\;M},{RT}_{C\;O},{RT}_{M\;O}\right). \end{array}$$ (30)

The left side above is the race outcome from four racers with their respective reaction times as *RT*_*CMO*_, *RT*_*C*_, *RT*_*M*_, and *RT*_*O*_, and the right side is the race outcome of another three racers with their respective reaction times. Since we are quite confident about the condition (that V1 lacks CMO cells) behind this equality, we call this a non-spurious race equality. It can quantitatively predict the distribution of *RT*_*CMO*_ from the distributions of the other six types of reaction times in the equality. Both the equality and its predicted *RT*_*CMO*_ distribution are also called non-spurious predictions.

Our derivation made clear that this equality does not depend on the details of the contextual influences in V1 other than its most prominent and essential aspects: iso-feature suppression that makes a feature singleton the most salient in our singleton scenes. Although important details such as colinear facilitation do play a role when asking other questions on saliency, as have been shown in model simulations and behavioral data [7, 30], the freedom of our non-spurious equality from such details makes our quantitative prediction possible. This is especially so since we do not yet have accurate information on these details [12, 13, 14, 17, 18, 19, 20, 22, 23] which may also depend on the observers (e.g., on their visual experience and adaptation states).

### The non-spurious prediction agrees with experimental data

**Fig 6:**
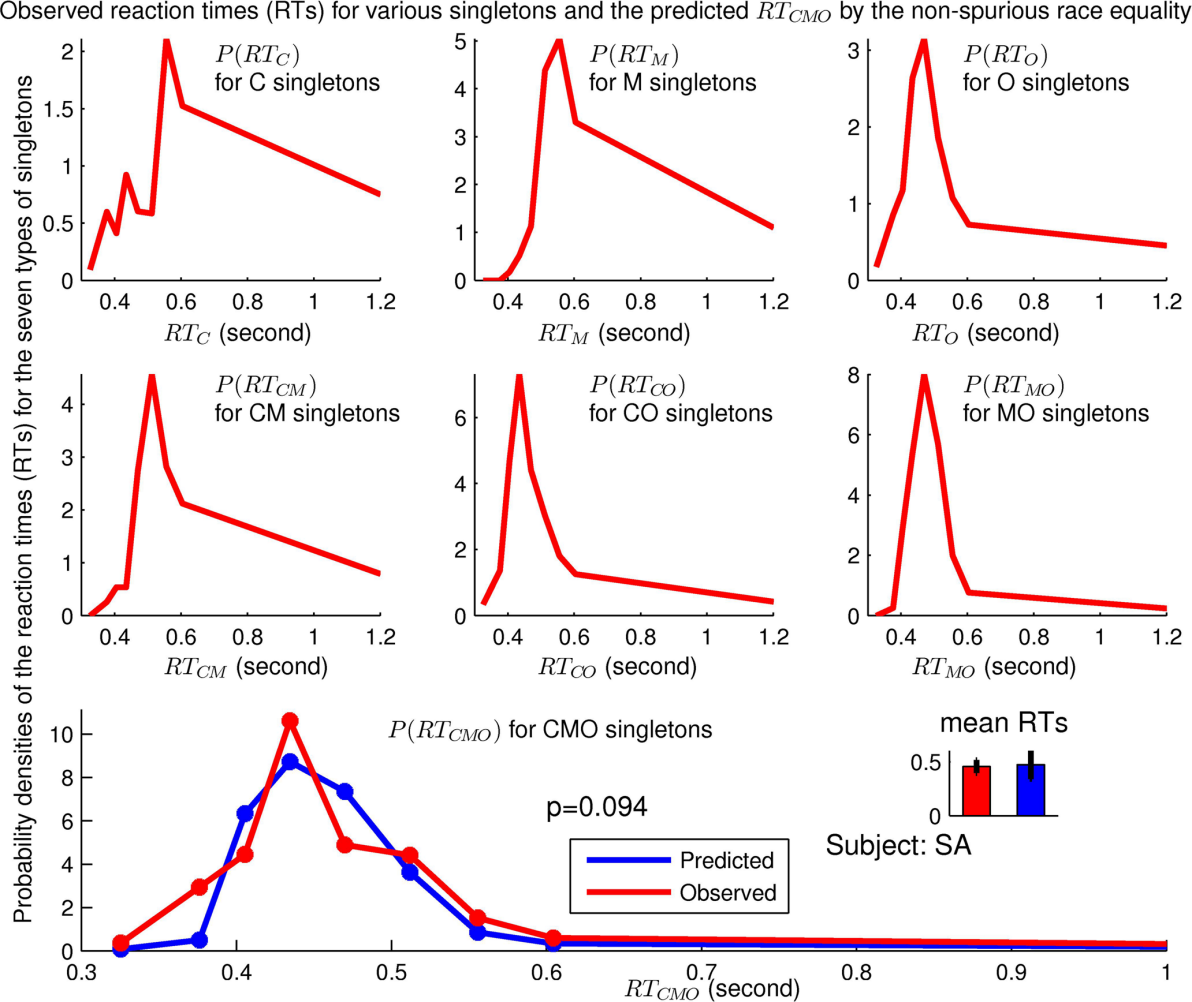
The observed distributions of *RT*_*C*_, *RT*_*M*_, *RT*_*O*_, *RT*_*CM*_, *RT*_*CO*_, and *RT*_*MO*_ for an observer are used to predict the distribution of *RT*_*CMO*_ for the same observer (SA who was also in Figs 3 and 5) by the non-spurious race equality $\text{min}({RT}_{C\;M\;O},{RT}_{C},{RT}_{M},{RT}_{O})\overset{P}{=}\text{min}({RT}_{C\;M},{RT}_{C\;O},{RT}_{M\;O})$. The predicted and observed distributions of *RT*_*CMO*_ are statistically indistinguishable from each other (*p* = 0.094). This figure has the same format as Fig 3.

**Fig 7:**
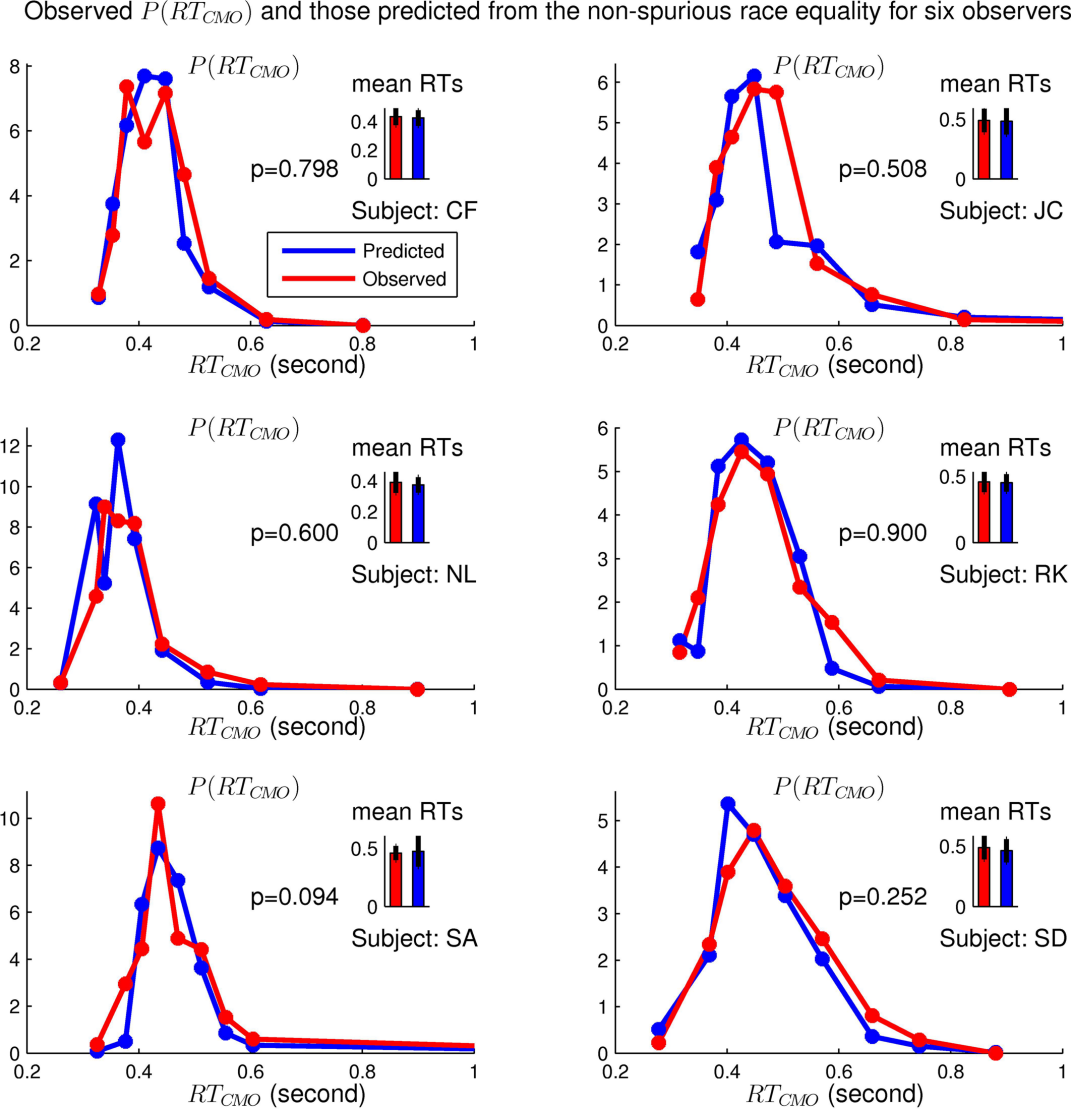
Observed and predicted distributions of *RT*_*CMO*_ using the non-spurious race equality for six observers, including observer SA whose details are shown in Fig 6. The predictions agree with data (indicated by *p* > 0.05) for all observers.

Fig 6 shows that the observed distribution of *RT*_*CMO*_ for our example observer SA is statistically indistinguishable from the non-spurious prediction using the other types of reaction times of this observer. Fig 7 shows that this agreement between the predicted and the observed *RT*_*CMO*_ holds for all six naive adult observers.

Is our non-spurious equality harder to falsify because it has a more complex structure than our spurious race models $RT_{\alpha \;\alpha^{\prime }}\overset{P}{=}\text{min}\left(RT_{\alpha },RT_{\alpha^{\prime }}\right)$ and $RT_{C\;M\;O}\overset{P}{=}\text{min}\left(RT_{C},RT_{M},RT_{O}\right)$? To answer this question, we create three new spurious equalities that are as complex as our non-spurious equality but can be falsified by the same data. Listing our non-spurious equality with these three newly created spurious equalities together,

$$\begin{array}{ccc} \text{non-spurious}: & \;\;\;\text{min}({RT}_{C\;M\;O},RT_{C},RT_{M},{RT}_{O}) & \overset{P}{=}\;\;\;\text{min}\left({RT}_{C\;M},RT_{C\;O},{RT}_{M\;O}\right), \end{array}$$ (31)

$$\begin{array}{ccc} \text{spurious}: & \;\;\;\text{min}({RT}_{C\;M\;O},{RT}_{M},{RT}_{C\;O}) & \overset{P}{=}\;\;\;\text{min}\left({RT}_{C},{RT}_{O},{RT}_{C\;M},{RT}_{M\;O}\right), \end{array}$$ (32)

$$\begin{array}{ccc} \text{spurious}: & \;\;\;\text{min}({RT}_{C\;M\;O},{RT}_{C},{RT}_{M\;O}) & \overset{P}{=}\;\;\;\text{min}\left({RT}_{M},{RT}_{O},{RT}_{C\;M},{RT}_{C\;O}\right), \end{array}$$ (33)

$$\begin{array}{ccc} \text{spurious}: & \;\;\;\text{min}({RT}_{C\;M\;O},{RT}_{O},{RT}_{C\;M}) & \overset{P}{=}\;\;\;\text{min}\left({RT}_{C},{RT}_{M},{RT}_{C\;O},{RT}_{M\;O}\right), \end{array}$$ (34)

we examine their similarities and relationships. For example, the left side of Eq (31) and that of Eq (32) are identical to each other if $RT_{C\;O}\overset{P}{=}\text{min}(RT_{C},RT_{O})$ holds, so are the right sides of the equations. Hence, Eq (32) is spurious when $RT_{C\;O}\overset{P}{=}\text{min}(RT_{C},RT_{O})$ is spurious, unless *RT*_*C*_, *RT*_*O*_, and *RT*_*CO*_ do not matter for the outcomes of their respective races (by being losers in the races), min(*RT*_*CMO*_, *RT*_*C*_, *RT*_*M*_, *RT*_*O*_) and min(*RT*_*CM*_, *RT*_*CO*_, *RT*_*MO*_), in the non-spurious equality. Similarly, the Eq (33) or Eq (34) is spurious when $RT_{M\;O}\overset{P}{=}\text{min}(RT_{M},RT_{O})$ or $RT_{C\;M}\overset{P}{=}\text{min}(RT_{C},RT_{M})$, respectively, is spurious, unless the corresponding racers are likely losers in the two races of the non-spurious equality. In other words, each of the three spurious equalities above is a corollary of a corresponding spurious (double-feature) race model $RT_{\alpha \;\alpha^{\prime }}\overset{P}{=}\text{min}\left(RT_{\alpha },RT_{\alpha^{\prime }}\right)$, which we refer to as the original spurious equality. Violation of the original spurious equality is necessary but not sufficient to violate its corollary equality (subject to random fluctuations in data samples).

**Fig 8:**
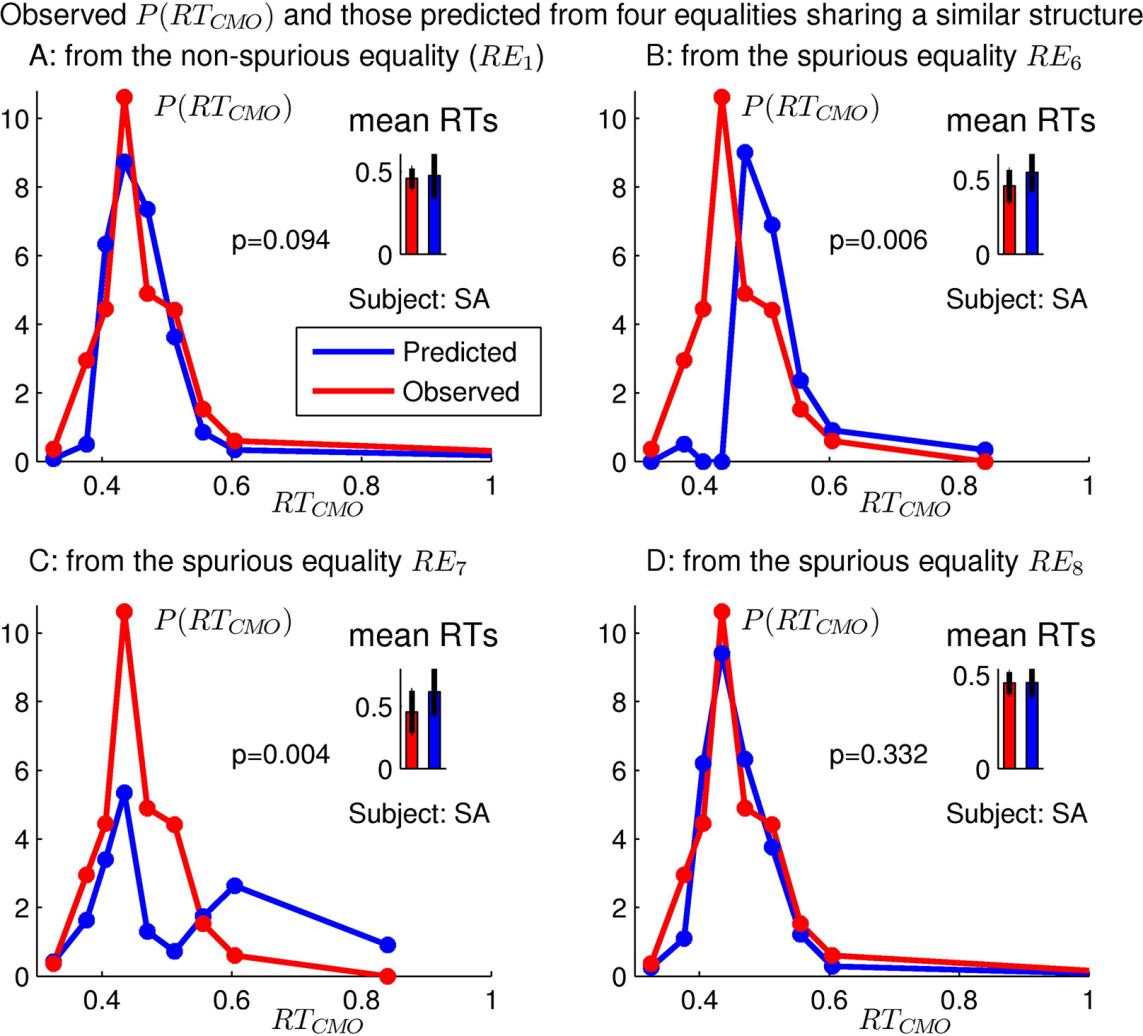
The predicted and observed *P*(*RT*_*CMO*_) from the non-spurious equality and the three spurious ones, listed in Eqs (31)–(34), are plotted in A, B, C, and D, respectively. These four equalities share a similar complexity and are also denoted as *RE*_1_, *RE*_6_, *RE*_7_, and *RE*_8_, respectively, in Table 1.

Each of Eqs (31)–(34) (one non-spurious) can predict the distribution of *RT*_*CMO*_ using the same set of six types of reaction times *RT*_*α*_ for *α* = *C*, *M*, *O*, *C* *M*, *C* *O*, *M* *O*. Fig 8B 8C 8D show that, in our example observer SA, the first two but not the last one of the spurious, corollary, equalities are falsified, mirroring the falsification of the original spurious equalities in Fig 5A 5B 5C. Hence, complexity in a race equality is insufficient to prevent its falsification.

### Qualitative conclusions across variations in the methods of data analysis

So far, we only illustrated the tests of the spurious equalities using data from one observer, and all the tests have so far been illustrated using a particular set of parameters characterizing the technical details in our procedures (see Methods) to test the race equalities. These technical details do not affect the qualitative conclusions. They can be parameterized by: (1) the number *N* of time bins to discretize the reaction time data samples for each singleton type of each observer, (2) the way to determine the boundaries between the time bins given *N*, (3) the metric to measure the distance *D* between the predicted and the observed distributions of the reaction times to judge whether a race equality holds, and (4) (only applicable to the four more complex equalities in Eqs (31)–(34)), the objective metric, i.e., the distance between the distributions on the two sides of a race equality, to be minimized in the optimization procedure to predict the *RT*_*CMO*_ distribution. The results presented so far in various figures are obtained using this set of parameters: (1) *N* = 9 (from one of five choices *N* = 8,9,10,11,12), (2) reaction time bins are chosen using Eq (45) with *x* = 1.35 (from four different choices listed around Eq (45)), (3) the *D* metric and (4) the objective metric are both the KL-like distance (the fourth of the four metric choices, see Eq (43)). This section presents some general statistics of our findings across 5 × 4 × 4 = 80 (or 5 × 4 × 4 × 4 = 320 for the more complex equalities) different sets of the parameters for the method.

Table 1 lists all the (spurious or non-spurious) race equalities, each in the format of $RT1\overset{P}{=}RT2$ with definitions of *RT*1 and *RT*2. For example, the equality $RT_{C\;O}\overset{P}{=}\text{min}(RT_{C},RT_{O})$ has *RT*1 ≡ *RT*_*CO*_ and *RT*2 ≡ min(*RT*_*C*_, *RT*_*O*_). Each race equality (RE) is indexed and referred to as *RE*_1_, *RE*_2_, …or *RE*_8_, for convenience. The *RE*_1_ is our (only) non-spurious equality $\text{min}({RT}_{C\;M\;O},{RT}_{C},{RT}_{M},{RT}_{O})\overset{P}{=}\text{min}({RT}_{C\;M},{RT}_{C\;O},{RT}_{M\;O})$. The *RE*_*i*_ for *i* = 2–4 are the double-racer models $RT_{\alpha \;\alpha^{\prime }}\overset{P}{=}\text{min}\left(RT_{\alpha },RT_{\alpha^{\prime }}\right)$ and the *RE*_*i*_ for *i* = 6–8 are their respective corollary (complex) equalities. The *RE*_5_ is the triple-racer model ${RT}_{C\;M\;O}\overset{P}{=}\text{min}\left({RT}_{C},{RT}_{M},{RT}_{O}\right)$. In each equality, the reaction time for the singleton with the largest number of unique features is designated (and denoted as *RT*_goal_ in Table 1) as the one whose distribution is predicted from those of the other reaction times. *RT*_*CMO*_ is the *RT*_goal_ for all race equalities except *RE*_*i*_ with *i* = 2–4, whose *RT*_goal_ are *RT*_*CO*_, *RT*_*MO*_, and *RT*_*CM*_, respectively. *RT*_goal_ tends to be the shortest reaction time in each equality, thus is more precisely determined, by the nature of the race(s), from the other reaction times.

**Table 1:** race equalities $RT1\;\overset{P}{=}\;RT2$ considered in this paper.

Equality Type/label	*RT*1	*RT*2	*RT*_goal_ designated for prediction
Non-spurious			
*RE*_1_	min(*RT*_*CMO*_, *RT*_*C*_, *RT*_*M*_, *RT*_*O*_)	min (*RT*_*CM*_, *RT*_*CO*_, *RT*_*MO*_)	*RT*_*CMO*_
Spurious			
*RE*_2_	*RT*_*CO*_	min (*RT*_*C*_, *RT*_*O*_)	*RT*_*CO*_
*RE*_3_	*RT*_*MO*_	min (*RT*_*M*_, *RT*_*O*_)	*RT*_*MO*_
*RE*_4_	*RT*_*CM*_	min (*RT*_*C*_, *RT*_*M*_)	*RT*_*CM*_
*RE*_5_	*RT*_*CMO*_	min (*RT*_*C*_, *RT*_*M*_, *RT*_*O*_)	*RT*_*CMO*_
*RE*_6_	min(*RT*_*CMO*_, *RT*_*M*_, *RT*_*CO*_)	min (*RT*_*C*_, *RT*_*O*_, *RT*_*CM*_, *RT*_*MO*_)	*RT*_*CMO*_
*RE*_7_	min(*RT*_*CMO*_, *RT*_*C*_, *RT*_*MO*_)	min (*RT*_*M*_, *RT*_*O*_, *RT*_*CM*_, *RT*_*CO*_)	*RT*_*CMO*_
*RE*_8_	min(*RT*_*CMO*_, *RT*_*O*_, *RT*_*CM*_)	min (*RT*_*C*_, *RT*_*M*_, *RT*_*CO*_, *RT*_*MO*_)	*RT*_*CMO*_

Koene and Zhaoping [29] collected reaction times for all the single- and double-feature singletons from eight observers, but collected *RT*_*CMO*_ data from only six of these observers. Hence, *RE*_*i*_ with *i* = 2–4 can be tested by eight observers while the other equalities by only six observers.

Whether a race equality can be falsified by data from a particular observer depends on several factors. First, as mentioned before, it may depend on the observer, as there may be inter-observer difference in terms of the V1 properties and visual sensitivities [44, 45]. Second, even when a race equality is truely false for a particular observer, it may appear to hold when there are insufficient samples of reaction time data, and thus insufficient statistical power in the data, to reveal a difference (particularly a small difference) between the prediction and its behavioral counterpart. Conversely, even when a race equality is fundamentally true, there is a 5% chance to find it accidentally broken by behavioral data. This is because, by definition (see Methods), a null hypothesis proclaiming the race equality is declared false when the distance *D* between the predicted (by the race equality) and observed distributions of reaction times is larger than 95% of the random samples of the distances *D* when the null hypothesis strictly holds. Third, empirically, the technical parameters (particularly the metric used to measure the difference between the predicted and observed distributions of reaction times) in our procedure can sometimes affect whether a race equality is falsified by data.

**Fig 9:**
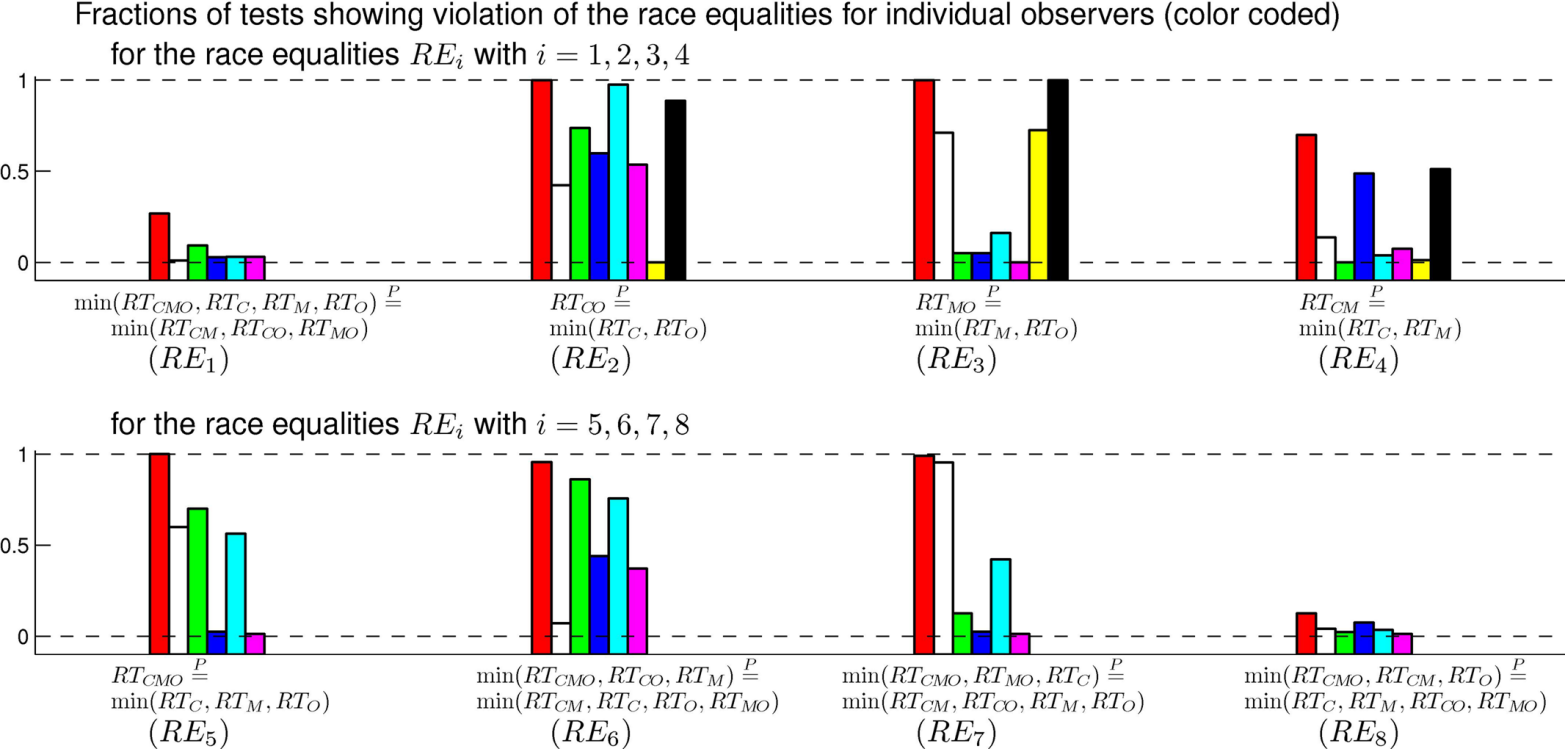
The fraction of the tests of each race equality that falsify the equality for each observer. Each observer is color coded by: red, white, green, blue, cyan, magenta, yellow, or black (red for our example observer SA). Different tests of an equality use different sets of parameters in the testing method to include all possible combinations of the parameter values. Each race equality is tested on six or eight observers as indicated. Results for *RE*_*i*_ for *i* = 2–4 is placed above that of its corollary equality *RE*_*i*+4_ for easy of comparison.

Fig 9 plots the fraction of all the (80 or 320) tests in which an equality is found broken in each observer and each race equality. In more than half of the cases, this fraction is either larger than 90% or smaller than 10%, indicating that the variations in the parameters of our method do not substantially affect whether the race equality holds. For some observers in some race equalities, e.g., observers marked by white, blue, and magenta color for *RE*_2_, a race equality is consistently broken using one metric and consistently maintained using another metric, (almost) regardless of the variations of the other parameters for the tests. For our non-spurious race equality, no test parameter value of any type consistently break the equality in any observer regardless of the other parameters.

Individual differences in neural response properties and a lack of statistical power in data are likely to partly explain why even the most obviously spurious equality ${RT}_{C\;O}\overset{P}{=}\text{min}({RT}_{C},{RT}_{O})$ is not broken by data from all observers. For example, the observer coded by yellow color in Fig 9 appears to show race equality ${RT}_{C\;O}\overset{P}{=}\text{min}({RT}_{C},{RT}_{O})$; this may either be caused by a lack of vigorously responding CO cells in this observer, or it may be because the difference between *RT*_*CO*_ and min(*RT*_*C*_, *RT*_*O*_) is too small to be detected by the limited number of random samples of each type of reaction times *RT*_*CO*_, *RT*_*C*_, and *RT*_*O*_.

Given a 5% chance to break a true race equality accidentally, there is a chance of $\frac{N!}{n!(N-n)!}0.05^{n}0.95^{N-n}$ that *n* out of *N* observers will break a true equality accidentally. Hence, if more than one or two out of six or eight observers, respectively, break a race equality, we say that the equality is broken or incorrect since such a high tendency of equality breaking can happen only by a chance of less than 0.05 for a truely correct race equality.

**Fig 10:**
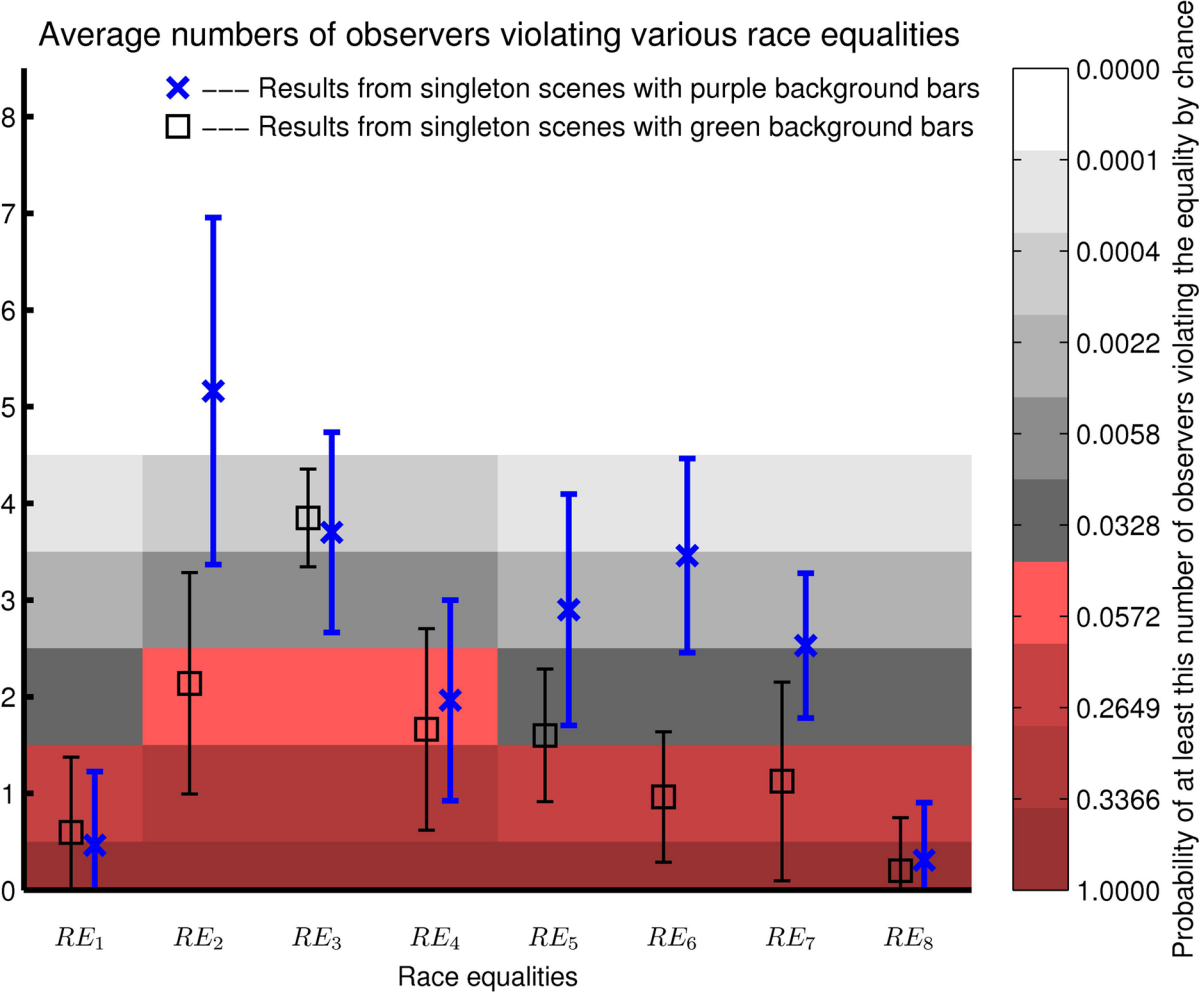
Average numbers of observers to break various race equalities, as shown in blue or black data points whose error bars denote standard deviations. The non-spurious race equality is *RE*_1_. Data from 6 observers were tested for race equalities *RE*_1_ and *RE*_*i*_ for *i* ≥ 5 and data from 8 observers were tested for *RE*_2_, *RE*_3_, and *RE*_4_. Applying a test of a given race equality to all the observers gives a number of observers breaking this equality, and the average of this number over 80 (for *RE*_*i*_ with *i* = 2–5) or 320 (for *RE*_1_ and *RE*_*i*_ with *i* > 5) tests, each characterized by a unique set of parameters in the testing method, gives a data point (blue cross or black square). The background shadings visualize the probabilities of at least a certain number of observers breaking a true race equality accidentally, shadings in red hue indicate probabilities larger than 0.05. Note that the number of observers in this probability representation is an integer number, whereas the data points are generally non-integers since they are averages of integer numbers.

Fig 10 plots the number of our observers to break each race equality, averaged over all the tests (each applied to all individual observers) which differ by the parameters in the testing method. Data points on gray or white background are those with more observers breaking an equality than expected by a probability of 0.05 if the equality truely holds. Blue crosses or black squares are, respectively, results from using *RT*_*α*_ data collected from purple or green scenes, respectively. Our results in Figs 3–9 are all based on data from the purple scenes. Focusing first on blue crosses (from purple scenes) in Fig 10, we have the following qualitative conclusions which are relatively immune to the sensitivities to the details in the testing method. First, the non-spurious race equality (*RE*_1_) is confirmed since it is only broken by an average of 0.5 observers, within the range expected for chance breaking of a true equality. Second, two spurious predictions, *RE*_2_ and *RE*_3_ (for equalities ${RT}_{C\;O}\overset{P}{=}\text{min}({RT}_{C},{RT}_{O})$ and ${RT}_{M\;O}\overset{P}{=}\text{min}({RT}_{M},{RT}_{O})$, respectively), are broken since data from more than about 3.5 observers break each of them, consistent with the presence of CO and MO neurons in V1 [34, 16]. Third, the spurious *RE*_4_ for equality ${RT}_{C\;M}\overset{P}{=}\text{min}({RT}_{C},{RT}_{M})$ is barely broken, or not as seriously broken as *RE*_2_ and *RE*_3_, since only around 2 out of eight observers have data violating it. This is consistent with the idea that V1 has fewer CM than CO or MO neurons, and is consistent with the controversy in experimental reports [46, 47, 48] regarding whether CM cells exist in V1. Fourth, the spurious prediction *RE*_5_ for equality ${RT}_{C\;M\;O}\overset{P}{=}\text{min}({RT}_{C},{RT}_{M},{RT}_{O})$ is broken since around three out of six observers violate it. This is consistent with the fact that V1 contains a substantial number of conjunctively tuned cells, in particular the CO and MO cells, and corroborates the finding that its component race equalities *RE*_2_ and *RE*_3_ are clearly broken. Fifth, the complex spurious equalities *RE*_*i*_ for *i* = 6–8, each a variation of the non-spurious *RE*_1_ and can be potentially undermined (when certain conditions hold, as discussed in the text around Eqs (31)–(34)) by the violation of the corresponding original *RE*_*i*−4_, are broken for *RE*_6_ and *RE*_7_ but maintained for *RE*_8_. This corroborates our findings for the original spurious *RE*_2−4_. The corollary equalities are less seriously broken than their original counterparts, lending further support to our non-spurious *RE*_1_ as it sustains the corollary equalities against the undermining factors from the violated original equalities.

However, more spurious predictions survive the test by data from the green scenes, see data points in black squares in Fig 10. In particular, the spurious $RT_{C\;O}\overset{P}{=}\text{max}(RT_{C},RT_{O})$ is only marginally broken. Reaction times for singletons unique in color, *RT*_*C*_, *RT*_*CO*_, *RT*_*CM*_, and *RT*_*CMO*_, tend be smaller in the green than purple scenes, particularly *RT*_*C*_ which is about 200–300 ms shorter in the green scenes. When both *RT*_*CO*_ and max(*RT*_*C*_, *RT*_*O*_) are closer to the minimum possible manual reaction time (around 0.3 second) of each observer, their difference also becomes smaller and is thus more difficult to be detected by the limited statistical power in our data. If we do a gross approximation by ignoring the difference between the green and purple scenes so as to increase the statistical power by pooling data across the two kinds of scenes, the outcomes are qualitatively the same as using data from the purple scenes alone except for *RE*_6_ which is marginally (rather than clearly) broken when data are pooled. Importantly, our non-spurious prediction *RE*_1_ agrees with data regardless of whether data come from the green or purple scenes.

The finding that the spurious equality *RE*_8_ agrees with our data is not a problem for the V1 saliency hypothesis. Recall that a prediction (or race equality) is called spurious in this paper if the neural properties upon which it relies are either uncertain or known to be violated in V1. If we were certain that V1 has no CM cells, then *RE*_8_ would be non-spurious since its original equality *RE*_4_ would be non-spurious. Hence, a marginally broken *RE*_4_ makes *RE*_8_ less likely broken, and the lack of serious violation of both *RE*_4_ and *RE*_8_ is consistent with the controversy regarding whether V1 has CM cells. If we were certain that V1 does have substantially responsive CM cells (such that $\left\langle r_{C\;M}^{C\;M}\right\rangle >\left\langle \text{max}(r_{C}^{C\;M},r_{M}^{C\;M})\right\rangle$ and $\left\langle r_{C\;M}^{C\;M}\right\rangle >\left\langle \text{max}(r_{C}^{C},r_{M}^{M})\right\rangle$) while *RE*_4_ is not substantially violated (given sufficient statistical power in data), then V1 saliency hypothesis would be falsified.

Our non-spurious *RE*_1_ and the spurious *RE*_*i*_ for *i* = 6–8 have very similar structures, they use the same technical procedure to predict *RT*_*CMO*_ from the same set of reaction times to the other singleton types. Hence, violations of equalities *RE*_6_ and *RE*_7_ suggest that our data have a sufficient statistical power in the purple scenes to reject our non-spurious equality *RE*_1_ if it were just as clearly incorrect as *RE*_6_ and *RE*_7_. Therefore, our non-spurious V1 prediction is confirmed within the resolution provided by the statistical power of our data. This resolution is manifested in Fig 8 in which it can clearly distinguish between the two reaction time distributions depicted in red and blue curves in Fig 8B or Fig 8C but not in Fig 8A or Fig 8D.

## Discussion

### The main finding

Our non-spurious prediction, $\text{min}({RT}_{C\;M\;O},{RT}_{C},{RT}_{M},{RT}_{O})\overset{P}{=}\text{min}({RT}_{C\;M},{RT}_{C\;O},{RT}_{M\;O})$, agrees with behavioral data such that the distribution of *RT*_*CMO*_ can be quantitatively predicted from those of the other types of reaction times of the same observer without any free parameters. This prediction is derived using the following essential ingredients: (1) the V1 saliency hypothesis that the highest V1 neural response to a location relative to the highest V1 responses to the other locations signals this location’s saliency, (2) the feature-tuned neural interaction, in particular iso-feature suppression, that depends on the preferred features of the interacting neurons to cause higher responses to feature singletons, (3) the data-inspired assumption that V1 does not have CMO neurons tuned simultaneously to color, motion direction, and orientation, and (4) the monotonic link (within the definition of saliency) between a higher saliency of a location and a shorter saliency-dictated reaction time to find a target at this location. Hence, our finding supports the direct functional link between saliency of a visual location and the maximum (rather than, e.g., a summation) of V1 neural responses to this location, as prescribed by the V1 saliency hypothesis. It also suggests that saliency computation (at least for our singleton scenes) essentially employs only the mechanisms with the following two properties: feature-tuned interaction between neighboring neurons (in particular iso-feature suppression) and a lack of CMO neurons, both available in V1, and neural mechanisms which are absent in V1 are not needed.

### The supporting findings

In addition, the following qualitative findings are obtained. First, two spurious predictions, ${RT}_{C\;O}\overset{P}{=}\text{min}({RT}_{O},{RT}_{O})$ and ${RT}_{M\;O}\overset{P}{=}\text{min}({RT}_{M},{RT}_{O})$, about which we have good confidence to be incorrect based on the V1 saliency hypothesis and the known presence of the CO and MO cells in V1, are falsified by our reaction time data. Second, using the V1 saliency hypothesis and our knowledge about the V1 neural substrates, we predicted relationships between the three predictions just mentioned, one non-spurious and two spurious, and the other five spurious predictions listed in Table 1. These relationships include the relative degrees of spuriousness between predictions and the dependence of some predictions on the non-spuriousness of some other predictions and certain properties of behavioral reaction times. The outcomes of testing the other five predictions are consistent with the predicted relationships, lending further support to the V1 saliency hypothesis.

### Implications for the V1 saliency hypothesis

Previously, the V1 saliency hypothesis provided only qualitative predictions. One example [21] predicts that an ocular singleton is salient and hence that the reaction time to find a visual search target is shorter when this target is also an ocular singleton, but it cannot quantitatively predict how much shorter this reaction time should be. Another example [30] predicts that a very salient border between two textures of oblique bars can be made non-salient (in a way unexpected from traditional saliency models) by superposing the textures with a checkerboard pattern of horizontal and vertical bars, but it cannot predict the quantitative increase in reaction times to locate the texture border by the superposing texture. Although these qualitative predictions are confirmed [21, 30], we cannot consequently conclude whether, in addition to the V1 mechanisms, more complex mechanisms available only in higher brain centers might also contribute to saliency computation. In contrast, if a prediction *quantitatively* specifies that one reaction time should be, say, 20% shorter than another one, and if data reveal instead that the first reaction time is only 10% shorter, then additional mechanisms for saliency computation must be called for. The quantitative agreement between our non-spurious prediction and the reaction time data without any free parameters suggests that saliency computation requires essentially no other neural mechanisms than those with the feature-tuned interactions between neurons and a lack of CMO neurons—both are V1 properties.

Let us articulate some other mechanistic ingredients or assumptions that were omitted in our closing sentence in the last paragraph and have been explicit or implicit in this paper. We assumed that the fluctuations in the responses of different types of neurons to an input item (e.g., responses of the C, O, and CO neurons to a CO singleton) are independent of each other. Also, fluctuations of the responses to different input items in a scene are assumed to be sufficiently independent of each other, so that we can treat the statistical properties of the responses to the background bars as independent of the responses to the singleton. We also assumed that the response of a neuron to a singleton is independent of whether this singleton is unique in a feature dimension to which this neuron is not tuned. For example, we assumed no statistical difference between $r_{C}^{C}$, $r_{C\;O}^{C}$, and $r_{C\;M\;O}^{C}$, between $r_{C}^{C\;O}$ and $r_{C\;M}^{C\;O}$, or between $r_{B}^{C}$ and $r_{O}^{C}$. This assumption may only be seen as an approximation given the known activity normalization in cortical responses [51]. Since V1 neurons’ responses are insensitive to small differences in luminance contrast when this contrast is very high [52], we also assumed that, when a V1 cell is not tuned to color, its response to our stimulus bar, which has a 100% luminance contrast against a dark background, is independent of whether our bar is green or purple, even though isoluminance between the two colors was not finely calibrated and adjusted to suit individual observers [29]. This assumption was needed to assume no statistical difference (e.g.,) between $r_{B}^{O}$ and $r_{C}^{O}$ and between $r_{O}^{O}$ and $r_{C\;O}^{O}$. The statistical properties of the population responses to the background bars are also assumed to be regardless of the type, location, and feature values of the singleton in our singleton scenes (provided that we restrict all singleton scenes to purple scenes only or to green scenes only, see Fig 4). This assumption enabled us to write Eq (3). Meanwhile, Eq (3) led to Eq (4) by an implicit assumption that fluctuations in the saliency readout to motor responses are negligible (this might be more likely for bottom-up than top-down responses). Furthermore, observers’ perceptual learning to do the visual search is assumed as negligible over the course of the data taking, so that the monotonic function relating V1 responses to reaction times is fixed. The above simplifications or idealizations were made to keep our question focused on the most essential mechanisms. That our prediction agrees quantitatively with data suggests that these simplifications or idealizations are sufficiently good approximations within the resolution that can be discerned by our data.

Future investigations could further test the V1 saliency hypothesis using more complex feature conjunctions. For example, one can test whether behavioral data on a conjunction of two orientations [30] match V1’s physiological property regarding whether V1 has sufficiently active cells tuned to such a conjunction [53].

### Implications for the role of extrastriate cortices

An important question is whether extrastriate cortices, i.e., cortical areas beyond V1, might also contribute to compute saliency. We have concluded that the two essential properties of the neural mechanisms for saliency computation are (1) feature-tuned contextual influences (in particular iso-feature suppression) and (2) a lack of CMO tuned cells. If extrastriate mechanisms also possess these properties, they could contribute to computing saliency, and we could extend to them the hypothesized link between the highest neural response to a location and the saliency of this location. After all, extrastriate visual areas also project to superior colliculus and so can influence eye movements.

Extrastriate cortices have been known [12] to exhibit feature-tuned contextual influences, in particular the iso-feature suppression. For example, V4 neurons exhibit iso-color, iso-orientation, and iso-spatial-frequency suppression [54, 55], V2 neurons exhibit iso-orientation suppression [56], and MT neurons exhibit iso-motion-direction suppression [12].

However, extrastriate cortices contain CMO neurons (private communication from Stewart Shipp, 2011). For example, Burkhalter and van Essen [35] observed that, in V2 and VP, many cells were feature selective in multiple feature dimensions, including orientation, color, and motion direction, and that the probability for a cell to be tuned in one feature dimension is independent of whether the cell is also tuned in another feature dimension. These observations imply that triple-feature tuned CMO cells are present. In fact, since they observed that most neurons are tuned to orientation and most neurons are tuned to color, the probability that a cell can be a CMO cell must be no less than 25% of the probability of this cell being tuned to direction of motion (M). Similar conclusions in V2 are reached by other investigations [57, 58], although different researchers use different criteria to classify feature tuning. In addition, unlike the case in V1 where the presence of CM neurons is controversial, V2 is known to have CM neurons in addition to CO and MO neurons [57, 48, 58]. Some of these CM, CO, and MO neurons (which are defined experimentally as being tuned to the two specified feature dimensions simultaneously without restrictions on the neuron’s selectivity in the other feature dimensions) in V2 can well be CMO neurons, especially when the chance for a neuron to be tuned to a feature dimension is independent of whether it is already tuned to any other dimensions. Selectivity to conjunctions of more than two types of features in extrastriate cortices is consistent with general observations that neurons in cortical areas beyond V1 tend to have more complex and specialized visual receptive fields.

According to our analysis in the Methods section, if a cortex containing the saliency map had CMO neurons, then, statistically, *RT*_*CMO*_ would be likely smaller than predicted by our non-spurious race equality $\text{min}({RT}_{C\;M\;O},{RT}_{C},{RT}_{M},{RT}_{O})\overset{P}{=}\text{min}({RT}_{C\;M},{RT}_{C\;O},{RT}_{M\;O})$, just as the presence of CO neurons makes *RT*_*CO*_ likely shorter than predicted by the race equality ${RT}_{C\;O}\overset{P}{=}\text{min}({RT}_{C},{RT}_{O})$. More specifically, our non-spurious equality was proven (in the Methods section) by using Eq (23) to write $\text{min}({RT}_{C\;M\;O},{RT}_{C},{RT}_{M},{RT}_{O})\overset{P}{=}f[\text{max}(Y)]$ and $\text{min}({RT}_{C\;M},{RT}_{C\;O},{RT}_{M\;O})\overset{P}{=}f[\text{max}(Y)]$, where *Y* is a list of responses from the single- and double-feature tuned neurons as specified in Eq (39). If CMO cells exist, then by Eq (23) four extra items $r_{C\;M\;O}^{C\;M\;O}$, $r_{C}^{C\;M\;O}$, $r_{M}^{C\;M\;O}$, and $r_{O}^{C\;M\;O}$ should be added to the list *Y* for min(*RT*_*CMO*_, *RT*_*C*_, *RT*_*M*_, *RT*_*O*_) and three extra items $r_{C\;M}^{C\;M\;O}$, $r_{C\;O}^{C\;M\;O}$, and $r_{M\;O}^{C\;M\;O}$ to the same list *Y* for min(*RT*_*CM*_, *RT*_*CO*_, *RT*_*MO*_). This upsets the equality unless either the CMO responses satisfy

$$\begin{array}{c} \text{max}(r_{C\;M\;O}^{C\;M\;O},r_{C}^{C\;M\;O},r_{M}^{C\;M\;O},r_{O}^{C\;M\;O})\overset{P}{=}\text{max}(r_{C\;M}^{C\;M\;O},r_{C\;O}^{C\;M\;O},r_{M\;O}^{C\;M\;O}), \end{array}$$ (35)

or if all CMO responses are negligible relative to max(*Y*), the maximum response of the list of single- and double-feature tuned neurons. Iso-feature suppression would typically make $r_{C\;M\;O}^{C\;M\;O}$ largest among $r_{\alpha }^{C\;M\;O}$ for all *α*, making $\left\langle \text{max}\left(r_{C\;M\;O}^{C\;M\;O},r_{C}^{C\;M\;O},r_{M}^{C\;M\;O},r_{O}^{C\;M\;O}\right)\right\rangle >\left\langle \text{max}\left(r_{C\;M}^{C\;M\;O},r_{C\;O}^{C\;M\;O},r_{M\;O}^{C\;M\;O}\right)\right\rangle$ likely so that *RT*_*CMO*_ is likely smaller than predicted unless the CMO responses are immaterial.

Assuming that the extrastriate CMO responses are not negligible and do not satisfy Eq (35), then the experimental confirmation of our non-spurious race equality suggests that, at least for our singleton scenes, extrastriate cortices contribute little to the guidance of exogenous attention (excluding the contribution to maintaining the state of alertness of observers). This suggestion is consistent with our previous finding [21] that an eye-of-origin singleton is very salient despite a paucity of eye-of-origin signals in every cortical area beyond V1.

Meanwhile, we do not know enough to rule out the possibility that the responses of the extrastriate CMO cells satisfy Eq (35) or are negligible relative to the responses from cells tuned conjunctively to fewer feature dimensions. For example, Eq (35) could hold if CMO responses could be invariant to any changes in the contextual inputs outside the classical receptive fields of these cells, in particular, if the extrastriate CMO responses could be exempted from the ubiquitous iso-feature suppression. The current study can hopefully motivate experimental investigations of the response properties of these extrastriate cells.

### Visual search in complex scenes, top-down factors in visual search, saliency in lower animal species, and representation of saliency in various brain regions

There remains an empirical question to ask if extrastriate cortices participate in saliency computation in more complex scenes. When top-down guidance is not held constant, one can no longer assume that reaction time (across different trials and scenes) relates monotonously with the saliency at a target’s location, making it difficult to test saliency hypotheses using reaction times. In a complex street scene for example, more than one saccade is typically required to search for, e.g., a person, whereas the singletons in our study can be typically located by the first saccade, leading to a manual reaction time to report the target less than a second in typical cases. Once the gist of a scene is comprehended within the first glimpse [59], the later saccades can be highly influenced by top-down knowledge [60, 61] (e.g., to direct gaze to the pavement but not the sky for finding a person). It is known that observers with and without object agnosia have very similar initial but not later saccades in viewing pictures [62], suggesting that initial saccades are relatively free from top-down factors via object-based knowledge [63]. Therefore, to answer our empirical question, we need more suitable measures than reaction times for a target not easily found by initial saccades. Meanwhile, having no neurons tuned to complex objects or features should not by itself exclude V1 from determining saliency in complex scenes. Most objects evoke V1 responses to their low level features, e.g., segments of a face contour. Such V1 responses could attract attention to objects before objects are recognized. A neural circuit model of V1 has showed that such responses could account for the fact that it is easier to find an ellipse among circles than a circle among ellipses [64, 7] and that angry faces tend to be more salient than happy ones [65].

Top-down factors can also affect short reaction times through expectation and goals. Krummenacher and Müller [66] showed that, CM singletons evoke a reaction time clearly shorter than predicted by the race model $RT_{C\;M}\overset{P}{=}\text{max}(RT_{C},RT_{M})$ from the V1 saliency hypothesis when assuming no CM cells in V1. However, they data taking blocks had the C, M, and CM singleton trials exclusively, the target was red and/or moving while the non-targets were always stationary and green, enabling top-down feature-based attention to red and/or moving bars. Furthermore, their search array had only 6 × 6 bars and the target was always within the central 4 × 4 bars, i.e., within the attentional window around the fixation at the start of a search trial (previous work [67] suggests that the attentional window size during visual search has a radius of about two in the units of average distances between neighboring search items), making it easier to exert top-down, goal-driven, target selection. (In contrast, our observers could not guess beyond chance the type, features, or location (which was always far beyond the central fixation) of the singleton in the next trial [29]). Their finding can thus be viewed as evidence supporting their idea of signal integration processes for a top-down (feature) dimension-weighting account [68, 69]. Indeed, feature-based, goal-directed, selections evoke enhanced responses in neurons in the frontal eye fields and V4 to visual inputs sharing the target’s features [70]. When they are useful for the task, repeated structures and details of visual inputs over trials can also guide attention [71] to contaminate behavioral measures for bottom-up saliency [72].

In lower animals like fish or frogs without a fully developed neocortex or V1, saliency computation is perhaps done in the retina or the optic tectum which is commonly called superior colliculus in mammals. Parallels of our saliency computation in singleton scenes are seen in archer fish preying on land-based insects by shooting them down with water [73]. The fish’s reaction time to attack a motion singleton, unique in speed, motion direction, or both features, is roughly independent of the number of preys in the singleton scene (but not in non-singleton scenes). Their tectum neurons exhibit iso-feature suppression in both feature dimensions of motion speed and motion direction, and some neurons are tuned conjunctively to both feature dimensions. Furthermore, the double-feature singletons attract attention more strongly while evoking stronger responses from the conjunctive cells. Hence, the V1 saliency map in primates may evolutionarily come from the tectum. It is of interest where the saliency map might be in animals such as rodents, whose V1 inputs to superior colliculus increase response magnitudes but not input selectivities of colliculus neurons [74].

As saliency affects behavior when read out for attentional shift (often combined with top-down factors for attentional guidance), it is unsurprising that neural correlates of saliency have been found in the superior colliculus [75] and in the parietal cortex [10, 76] and frontal eye field [77, 78], which also projects to the superior colliculus and are involved in top-down attentional control. In these downstream areas from V1 in the network for attentional control, saliency representation can be viewed as a copy or transformation of the saliency map in V1. For example, the map of graded saliency values can be transformed to a map of winner-take-all discrete values in which only the saccadic destination has a non-zero value. Indeed, in a color singleton search, the neural activities in superior colliculus [79], frontal eye field [80], and lateral intraparietal cortex [81] evolved from a map of activities at input locations of the search target and the non-targets to another map with activities merely or dominantly at the saccadic target destination. In the same vein, fMRI activities in the frontal eye field can be used to decode the most salient location in the visual field [78]. However, an explicit map of saliencies is computed and created in V1. Its content can be ignored, or combined with top-down factors, in the downstream areas such that the neural activities in all the three downstream areas are strongly affected by top-down, goal-directed, factors [77, 75, 76].

### Further discussions assuming no role in saliency by the extrastriate cortices

Although the current study cannot firmly establish the possibility that extrastriate cortices play no role in saliency, the implication of this possibility deserves pondering. The control of attentional selection, including exogenous selection, is traditionally thought to rest on a network of neural circuits comprising frontal and parietal areas [82, 10, 1]. The role of subcortical areas such as the superior colliculus has also been suggested [83]. An exclusion of extrastriate contributions from exogenous control should invite a fundamental revision of this network.

If exempted from guiding exogenous attention, extrastriate areas can focus on post-selectional decoding and/or endogenous selection [84]. Furthermore, in light of exogenous selection by V1, and since attentional selection admits only a tiny fraction of sensory information to be processed in detail, visual information processed in the extrastriate areas is likely to have a much smaller amount than that fed to V1 from the retina. This consideration should shape our investigations and shed light on some past observations. Indeed, unlike those in extrastriate areas, V1 activities are more associated with sensory inputs than with perception (i.e., outcomes of visual inference) and is less influenced by top-down attention [85]. For example, V4 lesions impair visual selection of only non-salient objects [86] disfavored by exogenous selection, demonstrating V4’s involvement in endogenous but not exogenous selection. Equally, neural responses in V4 but not V1 to binocularly rivalrous inputs are dominated by perceived input rather than the retinal images [87], contrasting V4 with V1 in perceptual decoding. Identifying V1’s role in exogenous selection thus helps to crystallize the research questions and pave the way for investigating extrastriate cortical areas.

## Methods

### Behavioral data to test various race equalities

We test various race equalities using data from Koene and Zhaoping [29]. Each of their stimuli contained 30 rows × 22 columns of bars (each randomly jittered from the regular grid location), extending about 39 × 29 degrees of visual angle. They collected about 300 samples of reaction times for each singleton category *α* = *C*, *M*, *O*, *C* *M*, *C* *O*, *M* *O*, or *C* *M* *O* from each observer, whose task was to press a left or right button, respectively, to report as quickly as possible whether a singleton was in the left or right half of the display, regardless of the feature(s) distinguishing the singleton. Each stimulus bar was a rectangle about 1 × 0.2^*o*^ in visual angle, took one of the two possible colors (green and purple), tilted from vertical in either clockwise or anticlockwise direction by a constant amount, and moved left or right at a constant speed, see Fig 4. All background bars were identical to each other in color, orientation, and motion direction; the singleton is unique in color, tilt direction, or motion direction, or any combination of these features. The green and purple colors had equal luminance (14 *cd*/*m* ^2^ in a black background) and equal color saturation in opposite CIE 1976 direction (hue angle 130^*o*^ and 310^*o*^, respectively) from neutral white at *u*′ = 0.2 and *ν*′ = 0.46. Given an observer, all bars had the same absolute angle from vertical, the same absolute motion speed, and the same color saturation; these absolute values were chosen for the observer and stayed fixed across all trials such that *RT*_*α*_ for each single-feature singleton *α* = *C*, *M*, or *O* was around 0.6 seconds on average (averaged across green and purple scenes). Different singleton scenes, in terms of the singleton type *α* and the color (green or purple), motion direction, and tilt direction of the background bars, were randomly interleaved. In each trial (of the data for this study), the singleton was randomly near 1 of 18 (9 left, 9 right) grid locations (in the 30 × 22 grid) at an eccentricity around 12.8^*o*^ from the display center where observers fixated at the start of the trial.

Trials with incorrect button presses or with reaction times shorter than 0.2 seconds or longer than three standard deviations above the average reaction time (for the particular observer and singleton type) were excluded from data analysis. Two out of the eight observers (four of them male) lacked data on *RT*_*CMO*_ (since they completed only an earlier version of the experiment). More details about the experiment can be found in the original paper [29], which did not publish or use the *RT*_*CMO*_ data. For each observer, data are divided into two pools, one collected from the green scenes and the other from the purple scenes; and each pool has about 150 *RT*_*α*_ data samples (on average) for each *α*. Results in Figs 3–9 are from analyzing data from purple scenes only. Fig 10 includes results from both types of scenes.

### Proof of the non-spurious race equality in Eq (30)

First, we use Eq (21) to write each *RT*_*α*_ in this equality as

$$\begin{array}{c} RT_{\alpha }=f\left[\text{max}\left(\text{list}\;\text{of}\;\text{non—trivial}\;\text{neuron}\;\text{responses}\;\text{to}\;\text{the}\;\text{singleton}\;\alpha \right)\right]. \end{array}$$ (36)

This generalizes Eq (20) to six types of V1 neurons *X* = *C*, *M*, *O*, *C* *M*, *C* *O*, and *M* *O*, of which none tuned to CMO, and to seven types singletons *α* = *C*, *M*, *O*, *C* *M*, *C* *O*, *M* *O*, and *C* *M* *O*. The response of neuron type *X* to singleton type *α* is $r_{\alpha }^{X}$. For example, by Eq (4) and analogous to Eq (17),

$$\begin{array}{c} {RT}_{C}=f[\text{max}(r_{C}^{C},r_{C}^{M},r_{C}^{O},r_{C}^{C\;M},r_{C}^{C\;O},r_{C}^{M\;O})]\overset{P}{=}f[\text{max}(r_{C}^{C},r_{B}^{M},r_{B}^{O},r_{C}^{C\;M},r_{C}^{C\;O},r_{B}^{M\;O})]. \end{array}$$ (37)

For the above, we used $r_{C}^{M}\;\overset{P}{=}\;r_{B}^{M}$, $r_{C}^{O}\;\overset{P}{=}\;r_{B}^{O}$, and $r_{C}^{M\;O}\overset{P}{=}r_{B}^{M\;O}$ which, analogous to Eqs (15)–(16), arise because a neuron’s responses to a singleton and a background bar are statistically the same unless the singleton is unique in at least one the feature dimensions to which this neuron is tuned. Then, keeping only the non-trivial responses to the C singleton, we get

$$\begin{array}{c} {RT}_{C}=f[\text{max}(r_{C}^{C},r_{C}^{C\;M},r_{C}^{C\;O})]. \end{array}$$ (38)

Analogously,

$$\begin{array}{ccc} {RT}_{M} & = & f[\text{max}(r_{M}^{C},r_{M}^{M},r_{M}^{O},r_{M}^{C\;M},r_{M}^{C\;O},r_{M}^{M\;O})]=f[\text{max}(r_{M}^{M},r_{M}^{C\;M},r_{M}^{M\;O})],\\ {RT}_{O} & = & f[\text{max}(r_{O}^{C},r_{O}^{M},r_{O}^{O},r_{O}^{C\;M},r_{O}^{C\;O},r_{O}^{M\;O})]=f[\text{max}(r_{O}^{O},r_{O}^{C\;O},r_{O}^{M\;O})]. \end{array}$$

Meanwhile,

$$\begin{array}{ccc} {RT}_{C\;M} & = & f[\text{max}(r_{C\;M}^{C},r_{C\;M}^{M},r_{C\;M}^{O},r_{C\;M}^{C\;M},r_{C\;M}^{C\;O},r_{C\;M}^{M\;O})]\\ & \overset{P}{=} & f[\text{max}(r_{C}^{C},r_{M}^{M},r_{B}^{O},r_{C\;M}^{C\;M},r_{C}^{C\;O},r_{M}^{M\;O})]=f[\text{max}(r_{C}^{C},r_{M}^{M},r_{C\;M}^{C\;M},r_{C}^{C\;O},r_{M}^{M\;O})]. \end{array}$$

The second line above used $r_{C\;M}^{C}\;\overset{P}{=}\;r_{C}^{C}$, $r_{C\;M}^{M}\;\overset{P}{=}\;r_{M}^{M}$, $r_{C\;M}^{O}\;\overset{P}{=}\;r_{B}^{O}$, $r_{C\;M}^{C\;O}\;\overset{P}{=}\;r_{C}^{C\;O}$, and $r_{C\;M}^{M\;O}\;\overset{P}{=}\;r_{M}^{M\;O}$, again because a neuron equates a unique feature with a background feature unless the neuron is tuned in this feature dimension. Analogously,

$$\begin{array}{ccc} {RT}_{C\;O} & = & f[\text{max}(r_{C\;O}^{C},r_{C\;O}^{M},r_{C\;O}^{O},r_{C\;O}^{C\;M},r_{C\;O}^{C\;O},r_{C\;O}^{M\;O})]\overset{P}{=}f[\text{max}(r_{C}^{C},r_{O}^{O},r_{C}^{C\;M},r_{C\;O}^{C\;O},r_{O}^{M\;O})],\;\text{and}\\ {RT}_{M\;O} & = & f[\text{max}(r_{M\;O}^{C},r_{M\;O}^{M},r_{M\;O}^{O},r_{M\;O}^{C\;M},r_{M\;O}^{C\;O},r_{M\;O}^{M\;O}])\overset{P}{=}f[\text{max}(r_{M}^{M},r_{O}^{O},r_{M}^{C\;M},r_{O}^{C\;O},r_{M\;O}^{M\;O})]. \end{array}$$

Similarly,

$$\begin{array}{c} {RT}_{C\;M\;O}=f[\text{max}(r_{C\;M\;O}^{C},r_{C\;M\;O}^{M},r_{C\;M\;O}^{O},r_{C\;M\;O}^{C\;M},r_{C\;M\;O}^{C\;O},r_{C\;M\;O}^{M\;O})]\overset{P}{=}f[\text{max}(r_{C}^{C},r_{M}^{M},r_{O}^{O},r_{C\;M}^{C\;M},r_{C\;O}^{C\;O},r_{M\;O}^{M\;O})]. \end{array}$$

Non-trivial responses to each singleton are listed under the scene schematics in Fig 4.

Using six types of V1 neurons (C, M, O, CM, CO, MO) instead of three types of V1 neurons (C, O, CO), one can generalize the derivations in Eqs (17)–(22) to verify that the race equality ${RT}_{C\;O}\overset{P}{=}\text{min}({RT}_{C},{RT}_{O})$ still does not hold in general.

Now, we apply Eq (23) to the left-hand side of our non-spurious equality,

$$\begin{array}{ccc} & & \text{min}({RT}_{C\;M\;O},{RT}_{C},{RT}_{M},{RT}_{O})\\ & & \;\overset{P}{=}f[\text{max}(r_{C}^{C},r_{C}^{C},r_{O}^{O},r_{O}^{O},r_{M}^{M},r_{M}^{M},r_{C}^{C\;M},r_{M}^{C\;M},r_{C\;M}^{C\;M},r_{C}^{C\;O},r_{O}^{C\;O},r_{C\;O}^{C\;O},r_{M}^{M\;O},r_{O}^{M\;O},r_{M\;O}^{M\;O})]. \end{array}$$ (39)

The list of the arguments in the *f*[max(…)] above is the collection of all the non-trivial neural responses to the corresponding singletons. Similarly, writing min(*RT*_*CM*_, *RT*_*CO*_, *RT*_*MO*_) from the right-hand side of our non-spurious equality as $\text{min}({RT}_{C\;M},{RT}_{C\;O},{RT}_{M\;O})\overset{P}{=}f\left[\text{max}(...)\right]$ gives the same list of arguments in *f*[max(…)] as in the equation above, thus proving the race equality.

In the list of arguments in *f*[max(…)] of Eq (39), each of $r_{C}^{C}$, $r_{O}^{O}$ and $r_{M}^{M}$ occurs twice as independent random samples. The list should not be simplified by deleting the repetitions, since the maximum of two random samples differs statistically from one random sample alone.

### Methods to test a race equality as a null hypothesis

**Fig 11:**
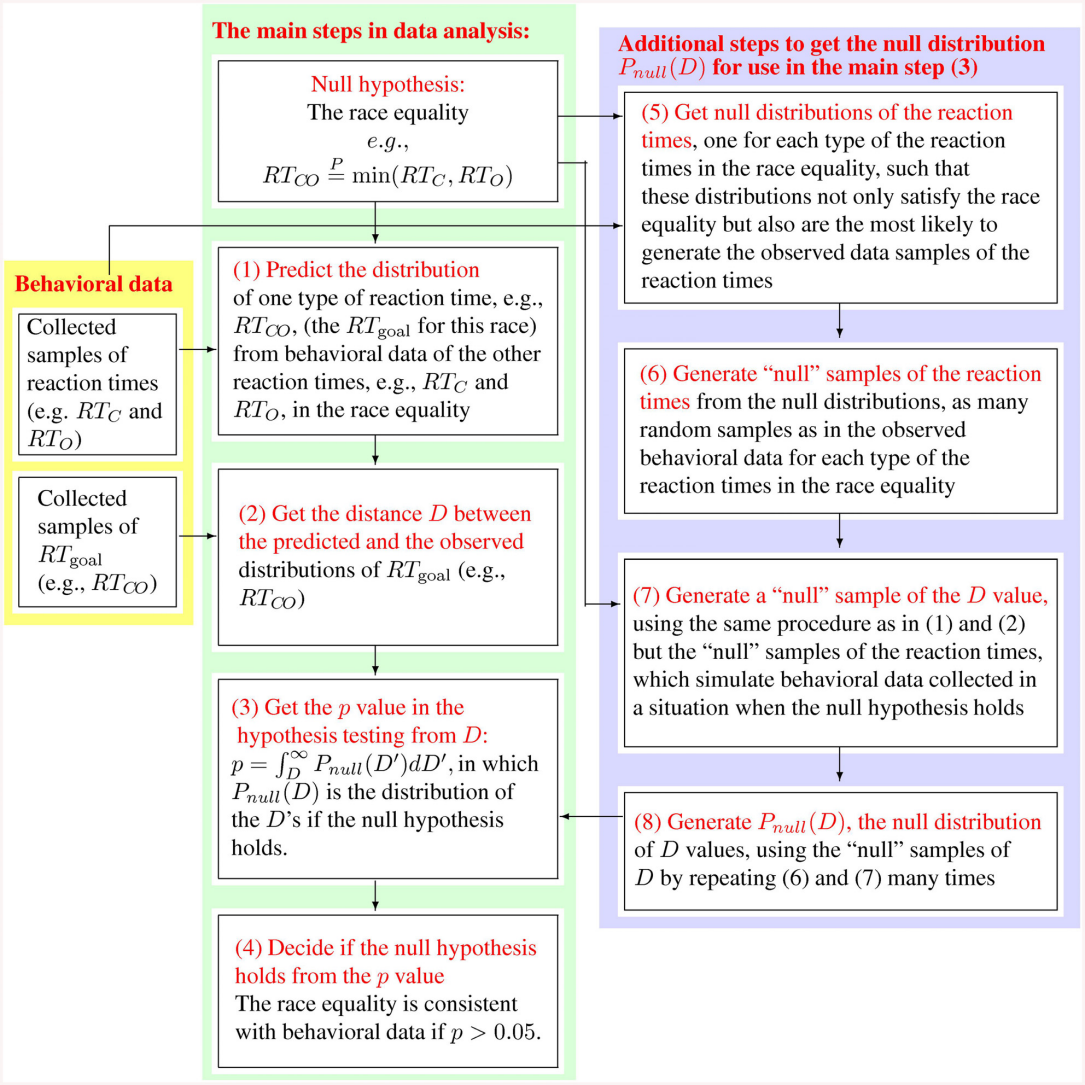
Diagram outlining the methods to test each of our race equalities, e.g., $RT_{C\;O}\overset{P}{=}\text{min}(RT_{C},RT_{O})$. The details of various components, in boxes (1)-(8), are described in the text.

Briefly, a race equality, e.g., $RT_{C\;O}\overset{P}{=}\text{min}(RT_{C},RT_{O})$, is a null hypothesis. It is used to predict the distribution of *RT*_goal_, the designated type of reaction times in the equality (e.g., *RT*_*CO*_ is the *RT*_goal_ for $RT_{C\;O}\overset{P}{=}\text{min}(RT_{C},RT_{O})$), from the behaviorally observed distributions of the other reaction times in the equality. A distance *D* is then calculated between the predicted distribution and the behaviorally observed one of *RT*_*goal*_. Typically *D* is non-zero even when a race equality does hold, since finite numbers of data samples can only approximately represent the underlying distributions of various reaction times. A statistical test is devised to give a *p* value, the probability that the *D* should be at least as big as observed if the null hypothesis holds. A *p* > 0.05 is chosen to suggest that the race equality agrees with data. The details of the components of the hypothesis testing method, represented by the boxes in Fig 11, are described next.

#### Methods to predict a distribution of reaction times from a race equality *RE*_*i*_

Here we describe the details for box (1) of Fig 11. First, given a race, e.g., min(*RT*_*C*_, *RT*_*O*_), min(*RT*_*C*_, *RT*_*M*_, *RT*_*O*_), or min(*RT*_*CMO*_, *RT*_*C*_, *RT*_*M*_, *RT*_*O*_), the samples of the winner of the race are

$$\begin{array}{ccc} & & \;\text{winner}\;\text{samples}\;\text{of}\;\text{a}\;\text{race}=\text{the}\;\text{collection}\;\text{of}\;\text{the}\;\text{minimums,}\;\text{one}\;\text{from}\;\text{each}\;\text{of}\;\text{all}\\ & & \;\text{possible}\;\text{combinations}\;\text{of}\;\text{the}\;\text{reaction}\;\text{time}\;\text{data}\;\text{samples}\;\text{from}\;\text{the}\;\text{racers,} \end{array}$$ (40)

regardless of the number of racers. For example, *m* samples of *RT*_*C*_ and *n* samples of *RT*_*O*_ give us *m* × *n* min(*RT*_*C*_, *RT*_*O*_) samples from the *m* × *n* possible combinations of *RT*_*C*_ and *RT*_*O*_ samples.

Each of our race equalities is in the format of $RT1\overset{P}{=}RT2$, and a reaction time type, designated as *RT*_*goal*_ (listed in Table 1), in the equality is predicted from data samples of the other reaction times in the equality. In race equalities *RE*_*i*_ for *i* = 2–5, *RT*_goal_ is *RT*1 and is the reaction time of a double-feature or triple-feature singleton, its predicted distribution is that of the samples of the race winner *RT*2 using data samples for the corresponding single-feature singletons (using Eq (40)).

In equalities *RE*_1_ and *RE*_*i*_ for *i* = 6–8, the *RT*_goal_ is always *RT*_*CMO*_. We write *RT*1 ≡ min(*RT*_*CMO*_, *RT*_part_), where, for *RE*_1_, *RE*_6_, *RE*_7_, or *RE*_8_, respectively, *RT*_part_ is min(*RT*_*C*_, *RT*_*M*_, *RT*_*O*_), min(*RT*_*M*_, *RT*_*CO*_), min(*RT*_*C*_, *RT*_*MO*_), or min(*RT*_*O*_, *RT*_*CM*_). Use Eq (40) to obtain samples for *RT*_part_ and *RT*2 using behavioral data samples of *RT*_*C*_, *RT*_*M*_, *RT*_*O*_, *RT*_*CM*_, *RT*_*CO*_, and *RT*_*MO*_. Then, samples of *RT*_part_, *RT*2, and data samples of *RT*_*CMO*_ are discretized into the same *N* time bins bounded by time values *t*_0_ < *t*_1_ < … < *t*_*N*_. Different *t*_*i*_’s are (in most data analysis) roughly evenly spaced except for very small and large *t*_*i*_’s. *N* = 8–12 is chosen to give sufficiently many behavioral data samples in each bin while maintaining a sufficiently large *N* for building a distribution.

Let distribution of any reaction times in *N* time bins be represented by an *N*-dimensional vector whose *i* ^*th*^ component is *n*_*i*_/(∑_*j*_ *n*_*j*_), where *n*_*i*_ is the number of these reaction time samples in the *i* ^*th*^ time bin. Let vectors **P** ≡ (*P*_1_, *P*_2_, …, *P*_*N*_) and **Q** ≡ (*Q*_1_, *Q*_2_, …, *Q*_*N*_) denote such distributions of *RT*1 and *RT*2, respectively, and let **p** and **q** denote the distributions of *RT*_*CMO*_ and *RT*_part_, respectively. *RT*1 ≡ min(*RT*_*CMO*_, *RT*_part_) means

$$\begin{array}{c} P_{i}=p_{i}\left(1-\sum_{j\leq i}q_{j}\right)+q_{i}\left(1-\sum_{j\leq i}p_{j}\right)+p_{i}q_{i},\;\text{for}\;\text{all}\;i. \end{array}$$ (41)

Then $RT1\overset{P}{=}RT2$ means *P*_*i*_ = *Q*_*i*_, i.e.,

$$\begin{array}{c} p_{i}\left(1-\sum_{j\leq i}q_{j}\right)+q_{i}\left(1-\sum_{j\leq i}p_{j}\right)+p_{i}q_{i}=Q_{i},\;\text{for}\;\text{all}\;i. \end{array}$$ (42)

Given **q** and **Q** (obtained from samples of *RT*_part_ and *RT*2, respectively), solve for **p** from the above linear equation. If this solution satisfies the probability constraints *p*_*i*_ ≥ 0 and ∑_*i*_ *p*_*i*_ = 1, it is our predicted distribution for *RT*_*CMO*_. Otherwise (this can happen for example when *q*_*i*_ > *Q*_*i*_ for some *i* due to fluctuations in the limited data samples and/or due to a lack of the race equality in reality), the predicted **p** is chosen as the one that minimizes a distance between **P** and **Q** under the constraints *p*_*i*_ ≥ 0 and ∑_*i*_ *p*_*i*_ = 1 (through an optimization procedure, e.g., via the fmincon routine in MATLAB). The following four different distance measures (between **P** and **Q**) were separately tried:

$$\begin{array}{c} \begin{array}{cc} (1): & {\left|P-Q\right|}^{2},\;\;\text{the}\;\text{squared}\;\text{Hemming}\;\text{distance,}\\ (2): & \sum_{i}{\left(\sqrt{P_{i}}-\sqrt{Q_{i}}\right)}^{2},\;\;\text{the}\;\text{Hellinger}\;\text{distance,}\\ (3): & \sum_{i}\left|P_{i}-Q_{i}\right|,\;\text{the}\;\text{1—norm}\;\text{distance,}\;\text{and}\\ (4): & \sum_{i}\text{max}\left(Q_{i},\epsilon \right)\log\frac{\text{max}(Q_{i},\epsilon )}{\text{max}(P_{i},\epsilon )},\;\;\;\;\text{with}\;\text{a}\;\text{given}\;\epsilon \ll 10^{-100},\;\text{the}\;\text{KL—like}\;\text{distance.}\; \end{array} \end{array}$$ (43)

The last distance is the Kullback-Leibler divergence if all *P*_*i*_ and *Q*_*i*_ were larger than a very small *ε*.

The boundaries *t*_*i*_ for the *N* time bins are determined as follows. Given a subject and a race equality, all the behavioral reaction time samples of all the singleton types in this race equality are put into a single pool. They are divided into *L* ≫ *N* time bins (*L* = 100 was used), whose boundaries

$$\begin{array}{c} T_{0}<T_{1}<T_{2}<...<T_{L}, \end{array}$$ (44)

are such that all bins contain (as close as possible) an equal number of samples from this pool. For reasons that will be clear soon, each *t*_*i*_ is chosen from among these *T*_*i*_’s as follows. Let *RT*(max) and *RT*(min), respectively, denote the largest and smallest data samples of the collective pool of *RT*_goal_, *RT*2, and (for *RE*_1_ and *RE*_*i*_ for *i* = 6–8) *RT*_part_ data samples. Given (*T*_0_, *T*_1_, …, *T*_*L*_), *t*_0_ is the largest *T*_*j*_ smaller than *RT*(min) and *t*_*N*_ is the smallest *T*_*j*_ larger than *RT*(max). Then, let *RT*′(max) and *RT*′(min) denote the largest and smallest *RT*_goal_ data samples, respectively. If *RT*′(min) > *RT*(min) and the largest *T*_*j*_ smaller than *RT*′(min) is larger than *t*_0_, then this *T*_*j*_ is assigned to *t*_1_. If *RT*′(max) < *RT*(max) and the smallest *T*_*j*_ larger than *RT*′(max) is smaller than *t*_*N*_, then this *T*_*j*_ is assigned to *t*_*N*−1_. Depending on whether *t*_1_ and *t*_*N*−1_ have just been assigned, there are now *N*′ = *N* − 1, *N* − 2, or *N* − 3 of the unassigned *t*_*i*_, which will be assigned in ascending order to *τ*_1_ < *τ*_2_ < … < *τ*_*N*′_. Each *τ*_*i*_ is the *T*_*j*_ value not yet assigned to any *t*_*j*_ for any *j* and is closest to the value $\tau_{i}^{\prime }$ which is larger than a fraction *F*_*i*_ (with *F*_1_ < *F*_2_ < … < *F*_*N*′_) of the *RT*_goal_ data samples. We tried each of the following four ways to choose *F*_*i*_’s. One is *F*_*i*_ = *i*/(*N*′ + 1). The others are

$$\begin{array}{c} F_{i}=\frac{1}{2}(erf(-x+2x\frac{i-1}{N^{\prime }-1})+1), \end{array}$$ (45)

in which *erf*(⋅) is the error function and *x* is a parameter with value *x* = 1.25, 1.35, or 1.45.

#### The statistical test for the null hypothesis

The Kolmogorov-Smirnov test cannot be used to test whether *RT*1 samples and *RT*2 samples are generated from the same underlying distribution, because the samples of at least *RT*2 are not independently generated. The following describes the methods in boxes (2)-(8) of Fig 11 for testing whether the predicted and observed distributions of *RT*_goal_ arise from the same underlying entity.

Given an observer and a race equality, let **p** and $\tilde{\text{p}}$ be the *N*-dimensional vectors for predicted and observed distributions of *RT*_goal_, respectively, in our *N* time bins. The distance *D* between **p** and $\tilde{\text{p}}$ (for box (2) of Fig 11) is measured by one of the four distance metrics in Eq (43), substituting $\tilde{\text{p}}$ and **p** for **P** and **Q**, respectively.

To test whether $\tilde{\text{p}}$ and **p** are statistically the same, we generated *m* = 500 other, simulated, distances *D* (box (8) of Fig 11). Each simulated *D* is a “null” sample for box (7) of Fig 11. It is obtained from a set of simulated samples of reaction times collected from a simulated behavioral experiment in a hypothetical situation when the race equality holds while the simulated data samples resemble the real behavioral data samples in terms of their distributions. Given the fixed time boundaries *T*_0_ < *T*_1_ < *T*_2_ < … < *T*_*L*_ (Eq (44)) obtained from the real behavioral data, the procedure to obtain a (simulated) *D* value using the simulated data samples is the same as that when the real data samples are used. The *p* value of the statistical test (box (3) of Fig 11) is the fraction of the simulated *D* values that are larger than the real *D* value (obtained using the real behavioral data), a *p* < 1/*m* = 0.002 is given when this fraction is zero. Our predicted and observed distributions of *RT*_goal_ are said to be significantly different from each other, i.e., not arising from the same underlying entity, and we declare that the race equality is broken, when *p* < 0.05 (box (4) of Fig 11)

To obtain simulated samples of reaction times for a race equality (box (6) of Fig 11), we should have already constructed (detailed in the next paragraph) a set of probability distributions, called the null distributions (of box (5) in Fig 11), for the reaction times involved in this race equality. The null distributions satisfy the race equality while being most likely to be the underlying distributions from which the behaviorally observed samples of reaction times could be generated. For example, for equality $RT_{C\;O}\overset{P}{=}\text{min}(RT_{C},RT_{O})$, the null distributions include three distributions, one each for *RT*_*CO*_, *RT*_*C*_, and *RT*_*O*_, respectively. From each of these null distributions, as many simulated samples of reaction times as the corresponding real behavioral samples of reaction times (for the corresponding singleton type) are randomly generated.

The null distributions in box (5) of Fig 11 are constructed as follows. Given a subject and a race equality, the real *RT*_*α*_ samples for all the singleton types *α* in the equality are discretized into *L* time bins using time boundaries *T*_0_ < *T*_1_ < … < *T*_*L*_ in Eq (44). Let **n**_*α*_ ≡ [(*n*_*α*_)_1_,(*n*_*α*_)_2_, …, (*n*_*α*_)_*L*_], and (*n*_*α*_)_*i*_ is the number of *RT*_*α*_ samples in the *i* ^*th*^ time bin. The likelihood, or probability, that an underlying distribution ${\hat{\text{p}}}_{\alpha }\equiv ({\hat{p}}_{\alpha_{1}},{\hat{p}}_{\alpha_{2}},...,{\hat{p}}_{\alpha_{L}})$ over these bins is the generator of **n**_*α*_ is proportional to $\Pi_{i=1}^{L}{\left({\hat{p}}_{\alpha_{i}}\right)}^{n_{\alpha_{i}}}$, whose logarithm is $\sum_{i=1}^{L}n_{\alpha_{i}}\text{ln}{\hat{p}}_{\alpha_{i}}+\text{constant}$. We construct null distributions ${\hat{\text{p}}}_{\alpha }$, one for each singleton type *α* in the race equality, such that the total log-likelihood

$$\begin{array}{c} \sum_{\alpha }\sum_{i=1}^{L}n_{\alpha_{i}}\ln{\hat{p}}_{\alpha_{i}}+\text{constant} \end{array}$$ (46)

is maximized, subject to the constraints that the race equality $RT1\overset{P}{=}RT2$ (which takes the form like Eqs (41)–(42)) is satisfied by these ${\hat{\text{p}}}_{\alpha }$s and, for each *α*, $\sum_{i=1}^{L}{\hat{p}}_{\alpha_{i}}=1$ and ${\hat{p}}_{\alpha_{i}}\geq 0$. The resulting ${\hat{\text{p}}}_{\alpha }$’s obtained through an optimization procedure (e.g., using fmincon in MATLAB) were verified to satisfy the race equality and sufficiently resemble the respective histograms of behavioral data *RT*_*α*_.

When each ${\hat{\text{p}}}_{\alpha }$ is viewed through coarser time bins for predicting the *RT*_goal_ distribution, the race equality remains satisfied since the boundaries *t*_*i*_ for these coarser time bins were chosen from those *T*_*j*_’s for the finer time bins. Although irrelevant to our outcome, the null distributions over continuous time can be approximated by (for each *RT*_*α*_) a uniform probability density ${\hat{p}}_{\alpha_{i}}/(T_{i}-T_{i-1})$ within the time window [*T*_*i*−1_, *T*_*i*_) and zero outside (*T*_0_, *T*_*L*_).
